# An Amino-Thiophene Functionalized Metal–Organic Framework on Fabric for Selective Extraction, Recovery, and Passive Sampling of Gold Ions and Nanoparticles

**DOI:** 10.1021/acs.chemmater.5c01238

**Published:** 2025-09-02

**Authors:** Vasiliki Gouma, Eleni C. Makri, Evangelos K. Andreou, Emilia Buchsteiner, Gerasimos S. Armatas, Manolis J. Manos, Dimosthenis L. Giokas

**Affiliations:** † Department of Chemistry, 37796 University of Ioannina, GR-45110 Ioannina, Greece; ‡ School of Chemistry, 7486 University of St Andrews, St Andrews KY16 9AL, U.K.; § Rigaku Europe SE, Hugenottenallee 167, 63263 Neu-Isenburg, Germany; ∥ Department of Materials Science and Engineering, University of Crete, GR-70013 Heraklion, Greece

## Abstract

The steadily increasing use of gold and the limited gold reserves have instigated a significant research effort to develop materials with efficient gold recovery. However, most works focus only on the uptake of Au ions, ignoring Au nanoparticles, which are increasingly used in various applications and represent a critical source of Au. Furthermore, the reported gold recovery studies involve powder-form materials that cannot be easily retrieved after the sorption process, thus making them unattractive for real, large-scale applications. The present work reports a Zr­(IV) metal–organic framework (MOF) with a defective microporous structure and amino-thiophene functional groups, which exhibit strong interactions with Au species. The MOF was immobilized on cotton fabric via an in situ method to create an easily retrievable bulk sorbent composite, which was investigated in detail for its sorption properties toward Au ions and Au nanoparticles (AuNPs). The immobilized sorbent showed relatively fast sorption kinetics (<1 and 3 h for Au ions and AuNPs, respectively), impressive sorption capacities (883.5 and 43.4 mg Au/g for Au ions and AuNPs, respectively), capability for capturing AuNPs irrespective of their coating and size and high recovery (>85%) of gold either as ions or AuNPs from genuine water samples. The latter property allowed the use of the MOF-fabric sorbent for the development of the first passive sampling sorbent phase for the long-term monitoring or recovery of AuNPs from natural waters. Notably, the sorbent was highly effective for the selective recovery of Au (∼97%) from an electronic waste simulant under flow conditions. The reusability of the MOF, in a composite form with calcium alginate, as Au sorbent was demonstrated under flow conditions for several cycles, indicating the potential of the material for Au separation applications. Overall, the reported sorbent offers a practical and cost-effective means for efficiently recovering ionic and nanoparticle Au species from complex water and wastewater media.

## Graphical abstract

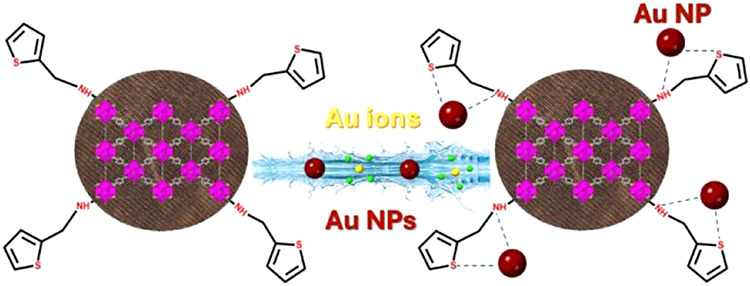

## 1 Introduction

Gold is a precious but nonrenewable resource that has been extensively mined and extracted since antiquity to produce jewelry and financial assets, such as currency. Since the beginning of the technological evolution in the 1940s, gold has been used to manufacture printed circuit boards, which are utilized in numerous electronic devices and currently account for approximately 7% of the global gold demand.^1^ This percentage is expected to increase in the coming years due to the significant growth of nanotechnology, which utilizes gold nanostructured materials in various products, including electronics, energy storage, paints, and biomedicine.

The increasing demand for gold may not only lead to the depletion of resources (the available known reserves of gold are estimated at 53 kilotons, which is almost half of the amount extracted since 1950),^2^ but also raises environmental concerns due to the mass production of electronic waste (e-waste) generated from electronic and electrical equipment. The improper disposal of such metal-rich e-waste results in a significant loss of economic resources (several billion euros)^3^ and may have adverse environmental effects due to the toxicity of gold salts and the presence of gold nanoparticles, which exhibit largely unknown ecotoxicity.^3^ Therefore, recycling e-waste and recovering gold species from industrial wastewater is economically and environmentally significant.

Gold recovery from leaching liquors and wastewater streams is feasible using various techniques such as solvent extraction, precipitation, hydrometallurgy, and sorption.^4–8^ Of these methods, sorption is considered a promising technique because it does not require any chemical reagents, it does not generate secondary waste and is cost-effective. However, conventional sorbents, such as carbon-based materials, ion-exchange resins, or biosorbents, show poor selectivity for gold, inadequate recyclability, and limited sorption capacity.^9–11^ For this reason, several advanced sorbents have been developed to uptake gold.^2,10,12–19^ Among these sorbents porous materials have garnered attention due to some unique advantages compared to other sorbents, including tailor-made functionality, high porosity, and a large surface area, which endow them with high uptake capacity, fast sorption kinetics, and good selectivity.^2,18^ However, several challenges are still associated with using porous sorbents (and other advanced sorbent materials) in realistic applications. These challenges concern the high cost of synthesis and their micro- or nanosized dimensions,^2,18,20^ which complicate their practical use, as they cannot be easily recovered, resulting in secondary pollution and higher application costs. The immobilization or incorporation of porous materials onto bulk supports, such as cellulose, fibers, fabrics, hydrogels, or aerogels, is a plausible approach to enhance the functionality of porous materials in practical applications by facilitating both their use and their recovery.^20^

Although significant progress has been made in the uptake and recovery of gold ions, gold nanoparticles (AuNPs), which are increasingly used in many industrial products and constitute a significant source of gold, have been largely overlooked. A limited number of sorbents have demonstrated the potential for capturing AuNPs through electrostatic interactions, chemisorption, and entrapment within their (macro)­pores.^21–27^ However, materials that enable the simultaneous uptake of dissolved gold ions and AuNPs are still scarce. To our knowledge, a Zr^4+^-mercaptosuccinate MOF with thiol groups forming strong covalent bonds with Au species,^21^ studied by our group, is the only sorbent reported to sorb Au ions and AuNPs. The sorbent demonstrated excellent performance in terms of sorption capacity and kinetics for Au ions and AuNPs under both batch and flow conditions. However, the easily oxidizable thiol moieties, which require specialized storage conditions (i.e., an inert atmosphere), raise concerns regarding the sorbent’s stability. In addition, the material has been tested in powder form, which is impractical for use in e-waste and wastewater streams. Not least, the Zr^4+^-mercaptosuccinate MOF was not reusable.

Considering the lack of published work on the field, we have rationally designed and fabricated a Zr^4+^-MOF with amino-thiophene functional groups [Zr_6_O_4_(OH)_4.64_(TA­TP)_4.03_(NH_2_­BDC)_0.64_(CH_3_­COO)_2.02_(H_2_O)_0.64_]·solvent (Metal Organic Resin-3, **MOR-3**, with TATP^2–^ = 2-thio­phene-2-yl­methyl)­amino)­tere­phthalate)), NH_2_BDC^2–^ = 2-aminoterephthalate) and immobilized it on cotton fabrics, via in situ coating, to create a freestanding sorbent (**MOR-3@pda­@cotton fabric**), which can conveniently be used in (waste)­water streams. The affixation of **MOR-3** on cotton fabric, instead of other materials such as paper or aero/hydrogels, imparts several advantages to its use as a receiving (sorbent) phase because: (a) cotton fabrics have improved mechanical and wetting stability compared to paper and gels,^28,29^ (b) sorption and mass transfer rates are enhanced due to the improved permeability of water through the large fabric pores and the high primary contact surface area of the cotton substrate,^29^ and (c) handling is facilitated, because cotton is a lightweight and flexible material that can be easily adjusted to various sizes and shapes, fitted to multiple devices, and quickly processed after use. Unlike the thiol-functional groups used in our previous work,^21^ thiophene exhibits high stability, while both thiophene and amine have a high affinity for Au species. The **MOR-3@pda­@cotton fabric** sorbent exhibited high sorption capacity (883.5 mg Au/g as AuCl_4_^–^ and 43.4 mg Au/g as AuNPs), decent uptake kinetics (<1 h for AuCl_4_^–^ and 3 h for AuNPs), and excellent selectivity. Significantly, Au can be recovered (>85%) using strong acidic or alkaline solutions. Based on experimental evidence, we unraveled the mechanism of gold recovery, which is primarily attributed to the chemical interaction of Au species with the thiophene and amine groups. Notably, the sorbent could effectively uptake ionic and particulate Au species from natural waters of variable matrix complexity . Capitalizing on this property, we have also developed the first passive sampling device for the long-term monitoring of AuNPs from environmental waters. Additionally, **MOR-3@pda­@cotton fabric** was utilized to recover gold from e-waste simulant under flow conditions. Although the MOF in its fabric composite is not reusable, the MOF in the form of calcium alginate beads exhibits exceptional reusability, as demonstrated by continuous flow adsorption–desorption studies. Overall, this work presents a holistic approach for the discovery of promising Au sorbents, involving the design, synthesis, and detailed characterization of the MOF with suitable functional groups, the subsequent immobilization of the MOF on a bulk substrate to create an easily retrievable sorbent and the thorough investigation of the immobilized sorbent for capturing Au species, either ions or nanoparticles, under a variety of conditions.

## 2 Experimental Section

### 2.1 Synthesis of 2-((Thio­phene-2-ylmethyl)­amino), Terephthalate (H_2_TATP)

The formation of the H_2_TATP ligand (Scheme S1) is based on the formation of a Schiff (imine) base between the amino group of 2-aminoterephthalic acid and the aldehyde group of 2-thiophene-carboxaldehyde, which in turn is reduced to form an amine. In detail, 2-thiophenecarboxaldehyde (1.5 mL, 16.5 mmol) was added to 60 mL of methanol in a conical flask containing 1.2 g (6.6 mol) of 2-aminoterephthalic acid and stirred for 2 h until a milky pale-yellow solution was formed. As a reductant (2.5 g, 66.08 mmol), solid sodium borohydride was added in small portions to avoid excessive bubbling. The conical flask was sealed, and the reaction proceeded overnight at room temperature under stirring. Then, 60 mL of diethyl ether was added to the solution, and a white precipitate was formed, which was isolated by filtration. The precipitate was dried (60–80 °C), redissolved in 50 mL of distilled water, and precipitated again by acid treatment by the dropwise addition of 6 M acetic acid until the pH of the solution was 5. The yellow organic ligand, 2-((thiophene-2-ylmethyl)­amino) terephthalic acid (H_2_TATP) was finally isolated by filtration, washed twice with 5 mL of distilled water, once with a mixture of 1:5 methanol/water, and dried at 60 °C for 24 h. The yield of this synthesis was 1.25 g.

### 2.2 Fabrication of **MOR-3@pda­@cotton Fabric**

A circular cotton fabric (4 cm diameter, 25.12 cm^2^ area on both sides, 25.75 cm^2^ surface area, 0.628 cm^3^ volume) was washed with acetone and distilled water to remove impurities and dried at 80 °C. The fabric was placed in a 20 mL Tris-base (10 mM) solution containing 0.05 g of 3-Hydroxy­tyraminium chloride and reacted overnight, under stirring. The polydopamine (PDA)-coated fabric was rinsed with distilled water and acetone to remove PDA that was not fixed in the fabric. The in situ synthesis and deposition of the MOF on the PDA-coated fabric was performed by the addition of the PDA-coated fabric into a 50 mL conical flask containing ZrCl_4_ (0.06 g, 0.257 mmol) and H_2_TATP­(0.1 g, 0.36 mmol) in 8 mL DMF/1.2 mL glacial acetic acid. The flask was sealed and heated at 85 ± 3 °C for 4 h to form an in situ deposit of the **MOR-3** particles on the surface of the PDA-coated fabric. The **MOR-3@pda­@cotton fabric** was washed sequentially with distilled water and acetone, dried at 80 °C, and stored in a desiccator until use. The amount of **MOR-3** deposited on the fabric was calculated by accurately weighing the fabric before and after deposition of **MOR-3**.

## 3 Results and Discussion

### 3.1 Synthesis and Characterization of **MOR-3** and **MOR-3@pda­@cotton Fabric**

The MOF was synthesized via a reaction of ZrCl_4_ and H_2_TATP in DMF and acetic acid, acting as a modulator, at 120 °C. Scanning Electron Microscopy (SEM) imaging revealed that the MOF particles are octahedral, with a size of 245 ± 4 nm (Figure S1a). Considering the high crystallinity and particle size (<500 nm), **MOR-3** was an excellent candidate for structural determination studies with microcrystal electron diffraction (MicroED, Figure S2).^30^ Due to the limited resolution of the MicroED technique^31^ and the positional disorder of the TATP^2–^-side groups, the precise structure of **MOR-3** cannot be determined; however, the overall structure connectivity can be correctly identified (Figure 1a). **MOR-3** crystallizes in the tetragonal space group *I*4/*m* with cell parameters *a* = 14.70(12) Å, *c* = 20.76(14)­Å, *V* = 4485(78) Å^3^. At first glance, the structure resembles a UiO-66 structure with Zr_6_O_4_(OH)_4_ clusters interconnected via terephthalate ligands (a total of 6 linkers), resulting in a 12-c framework (Figure 1a).^32^ Nevertheless, the organic ligands are not fully occupied, revealing the presence of missing linkers, i.e., a defective UiO-66 structure.^33^

**Figure 1:**
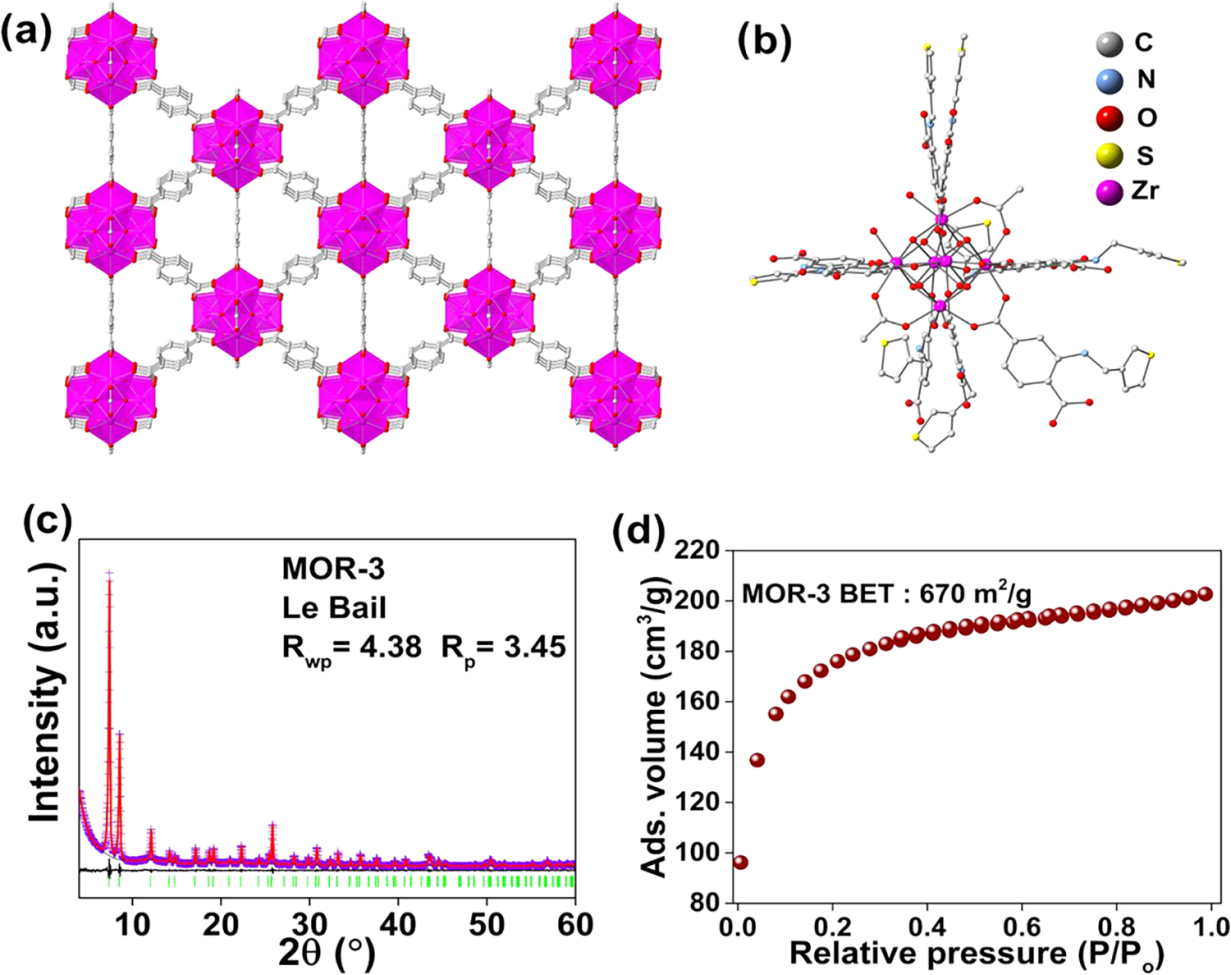
(a) Representation of the structure of MOR-3 determined from MicroED. (b) A structural model of the defective structure with missing linkers replaced by acetate and terminal water/hydroxide ligands. (c) Le Bail plot of MOR-3. Violet crosses: experimental points; Red line: calculated pattern; Black line: difference pattern (exp. – calc.); Green bars: Bragg positions. Refined unit cell parameters: *I*4/*m*, a = 14.678(2) Å, c = 20.797(4) Å, V = 4480(2) Å^3^. (d) N_2_ adsorption isotherm (77 K) of MOR-3.

Specifically, the refinement of the ligand occupancies (for details of the refinement, see Supporting Information) indicated 1.33 missing linkers, i.e., the total number of linkers is 6–1.33 = 4.67. Note that this is the first time that the number of missing linkers for a defective MOF is determined via MicroED, with the usual methods applied to compute defect sites being TGA or potentiometric titration.^33–35^ To elucidate the chemical formula of the MOF, we further used ^1^H NMR, TGA, and SEM-EDS data. Before the ^1^H NMR studies, a sample of the MOF was preheated at 150 °C. The heating process, aimed at removing most of the solvent molecules, was performed in the TGA apparatus, allowing us to relate the results from ^1^H NMR and TGA data directly. Then, the sample was digested in D_2_O/NaOH, and the ^1^H NMR spectrum was recorded. The data revealed a partial decomposition of the TATP^2^ ligand to NH_2_–BDC^2–^, which occurred during the MOF synthesis (a similar ligand ratio is obtained even when the sample is not preheated), with a molar ratio of TATP^2–^ to NH_2_–BDC^2–^ (as calculated from the peak integrals) equal to 1:0.16. Considering the determined ratio of linkers and the total number found from MicroED data (4.67), the TATP^2–^ and NH_2_–BDC^2–^ linkers are 4.03 and 0.64 per formula unit of **MOR-3**, respectively. In addition, the ^1^H NMR spectrum indicated the presence of 2.02 acetate ligands and 0.77 DMF residual solvent (Figures S4 and S5). The formula of a defective Zr^4+^ MOF with a monoanionic modulator, such as an acetate, can be written as Zr_6_O_4_(OH)_4+2x‑y_(L)_6‑x_­(modulator)_*y*_(H_2_O)_2x‑y_ (1), considering that missing linker sites not filled with modulator will be occupied by OH^–^ and H_2_O.^34^ However, at 150 °C, no lattice and coordinated water molecules are present (TGA, Figure S3). Thus, the formula of the MOF at this temperature, as calculated from the above general formula for x = 1.33 and y = 2.02 and the DMF-CH_3_COO content from the ^1^H NMR data, is Zr_6_O_4_(OH)_4.64_(TA­TP)_4.03_(NH_2_BDC)_0.64_(CH_3_­COO)_2.02_(DMF)_0.77_. The Zr content from this formula (26.19%) is close to that found from TGA data (Figure S3) for the water-free MOF (25.82%). Furthermore, EDS data indicated a Zr/S ratio of 6:3.7, relatively close to the calculated one (6:4.03) (Figure S6). Based on the general formula (1), the chemical composition of the MOF at room temperature, where coordinated water solvents are present, is [Zr_6_O_4_(OH)_4.64_(TA­TP)_4.03_(NH_2_BDC)_0.64_(CH_3_­COO)_2.02_(H_2_O)_0.64_]·solvent. In Figure 1b, a structural model of the MOF with 4.5 linkers per Zr_6_ unit, which is close to the calculated value (4.67) of linkers, is shown, illustrating the defective framework with acetate and terminal oxygen­(water/hydroxide) ligands replacing the missing linkers. In addition, the structural model contains mixed NH_2_BDC^2–^ and TATP^2–^ ligands with a ratio of 1:8 = 0.125, which is close to the experimental value (∼0.16). This model, derived from the structure determined from MicroED, was built and optimized using Avogadro.^36^ Additionally, the purity of the MOF was confirmed via PXRD and Le Bail analysis (Figure 1c). Stability study highlighted the retention of the MOF’s crystallinity in a wide pH range (0–11, Figure S7). ^1^ H NMR results indicated negligible modifications of the organic components in pH 2 and 10 and a somewhat increased NH_2_BDC^2–^ content in pH 11 (Figure S8), whereas after treatment with solutions with pH < 2, the TATP^2–^ to NH_2_BDC^2–^ ratio was significantly decreased, indicating an extensive transformation of the TATP^2–^ to amino-terephthalate ligand (Figure S9) under highly acidic conditions.

Nitrogen physisorption measurements for the activated **MOR-3** revealed a BET surface area of 670 m^2^/g (Figure 1d), which is relatively close to the BET surface areas of several Zr MOFs with terephthalate-based linkers and defective frameworks.^37,38^ Pore size distribution analysis indicated that **MOR-3** has a pore size of ∼5.2 Å, similar to that found for other Zr^4+^ MOFs with substituted-terephthalate ligands and defective frameworks (Figure S10d).^39^

The MOF was immobilized in the cotton fabric via an in situ coating method.^40^ Specifically, polydopamine (PDA)-functionalized fabric was added to the reaction mixture at 85 °C, where the formed MOF particles were deposited onto the fabric. PDA contains catechol functional groups that are excellent ligands for Zr^4+^, thereby allowing stronger binding of the MOF particles to the PDA-functionalized fabric as compared to plain fabric (Scheme S2). The mass of **MOR-3** deposited on the fabric by this coating method was estimated at 16 mg ± 1.8 per 25.12 cm^2^ of fabric. The successful immobilization of the MOF was confirmed via PXRD, SEM, and IR studies (Figure 2a–c).

**Figure 2:**
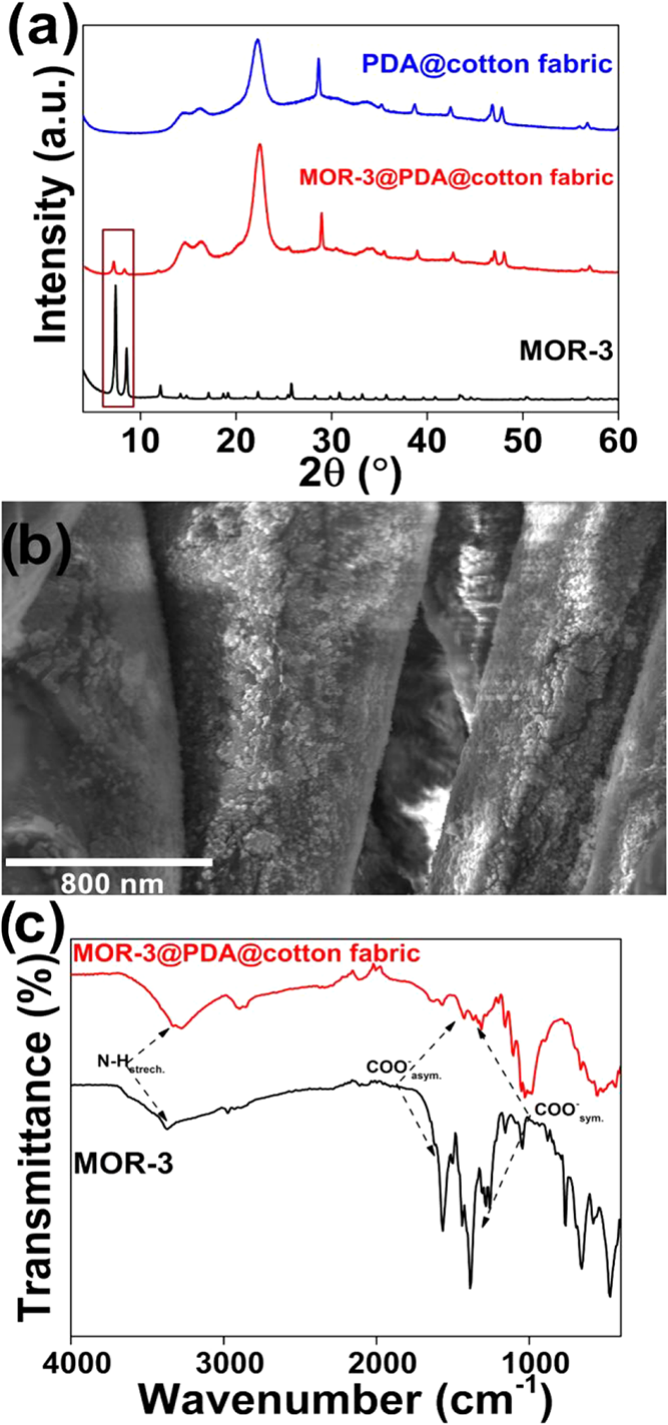
(a) PXRD patterns of MOR-3 powder compared to PXRD of MOR-3@pda@cotton fabric and PDA@cotton fabric. The characteristic diffraction peaks related to MOF are highlighted with a rectangle. (b) FE-SEM image and (c) IR data of MOR-3@pda@cotton fabric.

### 3.2 Sorption Studies of Au Ions and AuNPs

#### 3.2.1 Effect of pH

The influence of sample pH on the sorption of AuNPs and AuCl_4_^–^ ions was surveyed by adjusting the pH of the solutions from 3 to 8 using dilute HCl and NaOH solutions. Experiments were performed in 50 mL aqueous solutions containing each species separately to prevent coextraction. A semicircular **MOR-3@pda­@cotton fabric** was immersed in each sample and mixed at room temperature overnight in an orbital shaker at 150 rpm. The residual concentrations of AuNPs and Au ions in the solution were determined by atomic absorption spectroscopy (flame or graphite furnace, depending on the residual concentration of Au species).

The results of Figure S11 show that the **MOR-3@pda­@cotton fabric** exhibits some pH-dependent selectivity against AuNPs and AuCl_4_^–^ ions. At acidic pH values, both AuNPs and AuCl_4_^–^ ions are effectively extracted; however, at pH levels above ∼ 6, only Au ions can be efficiently removed by the sorbent, while the sorption efficiency for AuNPs deteriorates dramatically, from 85% to 10%. This finding indicates that coextraction may occur if both species are present in the sample, even to a lower extent for AuNPs. Considering that Au ions maintain their solubility at all pH values in the form of AuCl_*x*_(OH)_*y*_)^−^ (*x* + *y* = 4) complexes,^41^ the efficient capture of Au ions may be explained by their strong covalent interactions with the MOF’s amine and thiophene functional groups. Concerning AuNPs, the high sorption efficiency in acidic solutions may be attributed to the weakening of interactions between Au atoms and the capping agent, PVP. Under such conditions, the coordination capability of carbonyl oxygen atoms of PVP is hindered, thus allowing the interactions of Au species with the thiophene functional groups of the MOF. Instead, under neutral to basic conditions, the relatively strong binding of carbonyl-PVP groups to the Au species limits the sorption of AuNPs by the MOF.^42^

#### 3.2.2 Sorption Kinetics

Considering the influence of pH, the investigation of sorption kinetics was studied through batch sorption experiments at pH 6 for AuCl_4_^–^ ions (3 μg/mL), which is close to the pH of natural waters, and at pH 3 for PVP@AuNPs (5 nm, 3 μg/mL Au), which is close to the pH of most industrial wastewater and e-waste leachates, using a semicircular **MOR-3@pda­@cotton fabric** at variable time intervals (Figure 3a,b). Sorption experiments were performed separately for each Au species to avoid synergistic or competitive interactions. The sorption kinetics were then fitted to the pseudo-first order (PFO) and pseudo-second order (PSO) kinetic models (Figure 3a,b). The sorption kinetics of PVP@AuNPs could only be described by the Ho–McKay pseudo–second order (PSO) kinetic model, indicating that chemical sorption is the primary removal mechanism, attributed to the strong interactions of AuNPs with the MOF’s functional groups. Concerning AuCl_4_^–^ ions, fitting was satisfactory with both the HoMckay pseudo–second order (PSO) kinetic model (*R* ^2^ = 0.97) and the Lagergren’s pseudo–first order (PFO) model (*R* ^2^ = 0.99), indicating that the sorption of AuCl_4_^–^ ions is a multistage process, as will be discussed below.

**Figure 3:**
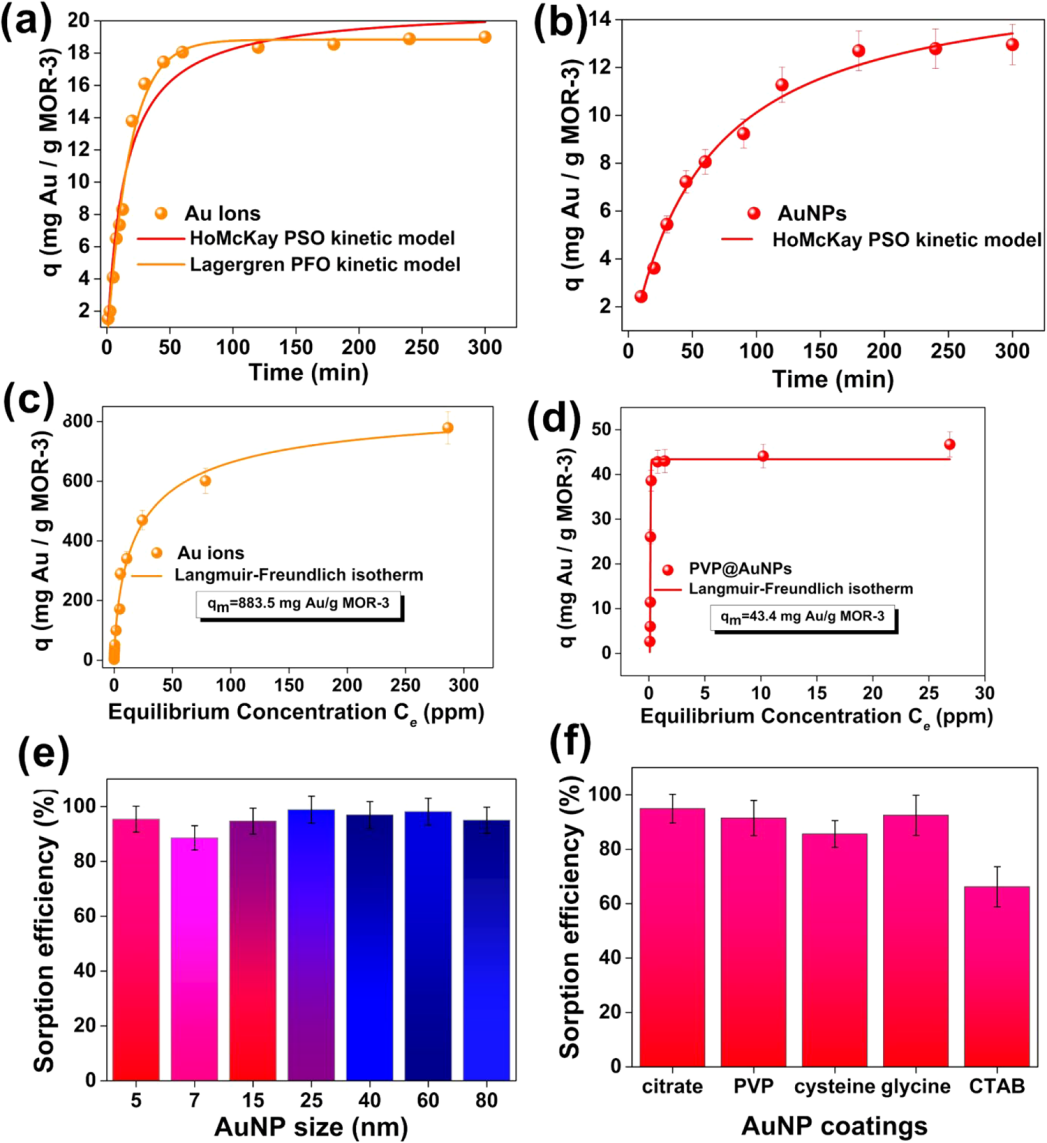
Fitting of the kinetic data with the Ho-Mckay’s pseudo-second-order equations (red lines) and Lagergren’s pseudo-first order (orange line) for the sorption of (a) Au ions, and (b) PVP@AuNPs by MOR-3@pda@cotton fabric. Sorption isotherms for (c) Au ions and (d) PVP@AuNPs by MOR-3@pda@cotton fabric, with the line representing the fitting with the Langmuir–Freundlich model. Sorption efficiency of MOR-3@pda@cotton fabric for the removal of AuNPs of different (e) sizes and (f) surface coating.

The kinetic data were also fitted to Weber and Morris’s intraparticle diffusion model (Figure S12), which shows multilinearity. This indicates that the adsorption mechanism follows a film diffusion pattern and involves more than one rate-limiting step, with intraparticle resistance playing a dominant role.

#### 3.2.3 Sorption Isotherms

We performed sorption isotherm studies of Au ions and AuNPs for the **MOR-3@pda­@cotton fabric** sorbent and compared the results with those obtained from **MOR-3** powder. Specifically, batch sorption isotherm studies were conducted at pH 3 for PVP@AuNPs (5 nm) and pH 6 for AuCl_4_^–^ ions (Figure 3c,d). The sorption of both PVP@AuNPs and AuCl_4_^–^ ions on both powder and fabric sorbents could be described by the Langmuir–Freundlich isotherm model (Tables S1 and S2), which accounts for both monolayer and multilayer adsorption on heterogeneous surfaces. The maximum sorption capacities of **MOR-3@pda­@cotton fabric**, calculated using the Langmuir–Freundlich model, were 43.4 ± 5.8 mg Au/g **MOR-3** for PVP@AuNPs (or 50.2 nmol PVP@AuNPs/g **MOR-3**) and 883.5 ± 63 mg Au/g **MOR-3** for AuCl_4_^–^ ions. In comparison, the maximum sorption capacities of PVP@AuNPs and AuCl_4_^–^ on **MOR-3** **powder** were 35.4 ± 8 mg Au/g **MOR-3** and 889 ± 44 mg Au/g **MOR-3**, respectively (Figure S13, Table S2). Thus, the sorption capacities of the powder and fabric sorbents are in good agreement, indicating that the sorption capability of the MOF material remains practically unchanged (within the error limits) when it is immobilized in the fabric. In addition, these results indicate that the sorption capacity of the **MOR-3@pda@cotton fabric** is due to the MOF, rather than the cotton fabric or PDA.

#### 3.2.4 Sorption of AuNPs of Various Sizes and Surface Coatings

AuNPs in industrial wastewater and the environment can be present in different sizes, morphologies, and surface coatings. Moreover, AuNPs in environmental media may undergo various morphological transformations and alterations in their surface properties.^43^ Therefore, a sorbent shall effectively uptake all AuNPs irrespective of size, shape, and surface functionalization. The efficiency of **MOR-3@pda­@cotton fabric** to remove AuNPs of different sizes and surface functionalization was evaluated using PVP10@AuNPs of various sizes (5–80 nm) and AuNPs (4 nm) coated with small molecules (citrate), polymers (PVP), thiols (cysteine), amino acids (glycine), and surfactants (CTAB). According to the results shown in Figure 3e, the sorption efficiency of **MOR-3@pda­@cotton fabric** for AuNPs of various sizes (5–80 nm) improved with increasing size and ranged from 88.6 to 98.8%, while the sorption of AuNPs with different surface coatings (Figure 3f) ranged from 85.6 to 95.0%, except for CTAB@AuNPs, which were removed by 66.2%. These data indicate that **MOR-3@pda­@cotton fabric** can effectively uptake AuNPs of different sizes and coatings, and hence, it is suitable for the sorption of total AuNPs in environmental samples.

### 3.3 Gold Capture and Recovery Applications

#### 3.3.1 Extraction of Gold Species from Natural Waters

The uptake of gold and gold nanoparticles from environmental samples is challenging due to the presence of various matrix components, including organic matter, inorganic salts, and microorganisms, which influence their fate and behavior in the environment.^43^ Moreover, at the typical pH values of most environmental waters (pH 6.5–8.0), the sorption of Au ions remains unaffected. In contrast, the sorption of AuNPs exhibits a dramatic decline (Figure S11), reaching approximately 10% of its maximum value, corresponding to a concentration of ≤4.3 mg/g. Considering that the measured environmental concentrations of gold ions and gold nanoparticles are at the ng/L level,^44–47^ the sorption capacity of **MOR-3@pda­@cotton fabric** at pH ≥ 6 would still be adequate for the uptake of AuNPs in natural waters. This value (4.3 mg AuNPs/g) may also provide an adequate sorption capacity in the worst-case scenario, where the predictions of probabilistic and material flow algorithms are verified, and the concentrations of AuNPs in natural waters increase to μg/L levels.^47–49^

To evaluate the potential of **MOR-3@pda­@cotton fabric** to collect Au ions and Au nanoparticles (AuNPs) from water matrices, various water samples with varying matrix complexity were fortified with a mixture of Au ions and PVP10@AuNPs at a concentration of 1 μg Au/mL. This concentration is lower than the maximum sorption capacity of the sorbent at all pH values and higher than the environmental concentration of Au species. The experimental setup used for this test involved the batch sorption of Au ions and AuNPs onto **MOR-3@pda­@cotton fabric** under stirring (i.e., fabric-phase sorptive extraction).^50^ The results depicted in Figure 4a show that the removal efficiency of **MOR-3@pda@cotton fabric** exceeded 85% in all water samples, indicating the potential of the sorbent to collect Au species from the most typical water matrices. It should also be noted that no leaching of Zr^4+^ ions and organics was found after treatment of water samples with **MOR-3@pda­@cotton fabric**. Specifically, the Zr content of the samples, as determined by ICP-OES, was not detectable (<LOD), while ^1^H NMR analysis showed no residues of DMF or the organic ligands (Figure S14).

**Figure 4:**
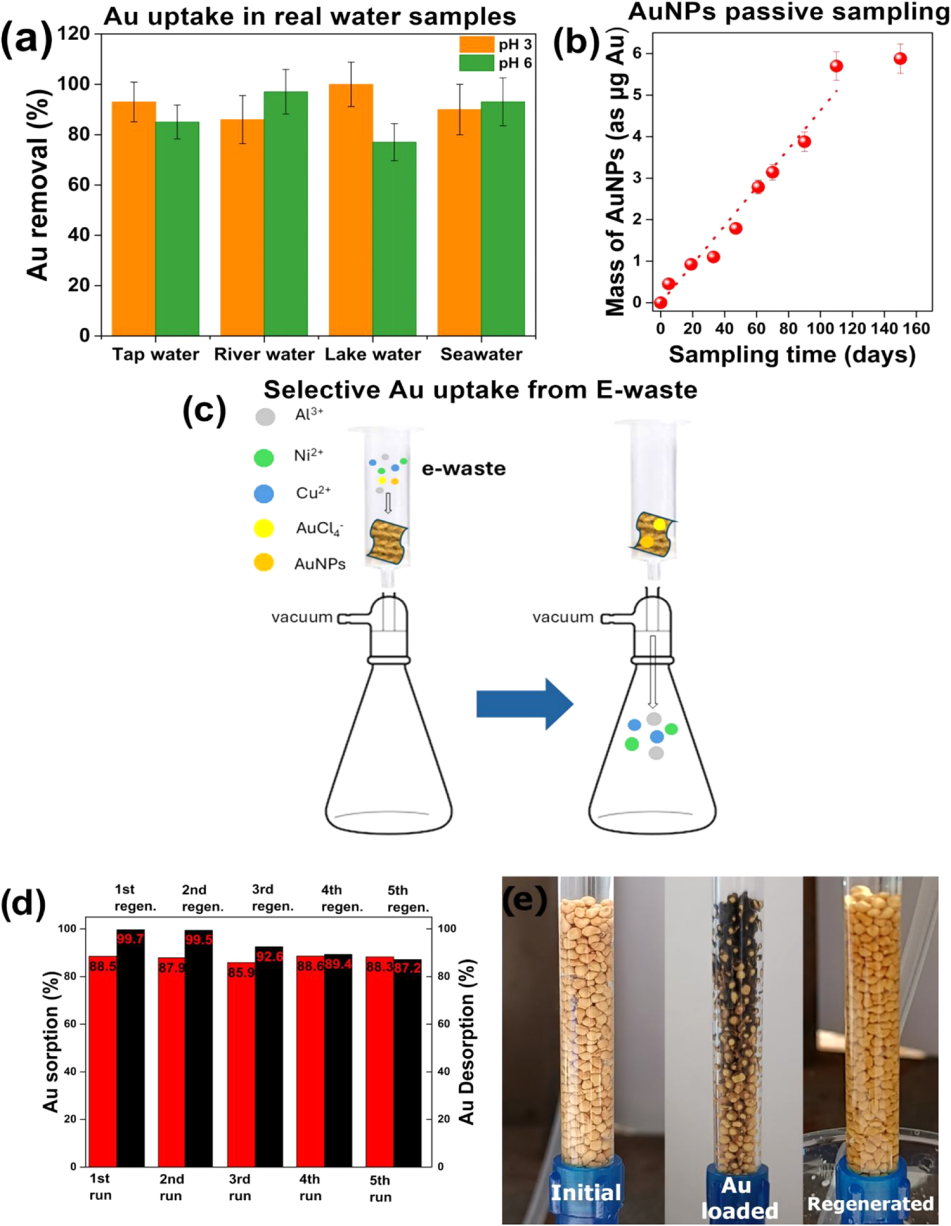
(a) Removal of Au species (AuCl_4_^–^ ions and AuNPs at a nominal Au concentration of 1 μg/mL) from various water samples. (b) Passive sampler (MOR-3@pda@cotton fabric) uptake curve for PVP@AuNPs. (c) A schematic of the apparatus used to treat the electronic waste simulant solution. (d) Au sorption and desorption percentages in each cycle. (e) MOR-3@CA beads before (left), after Au sorption (center), and after regeneration with thiourea solution (right).

#### 3.3.2 Passive Sampling

Motivated by these findings, we further explored the applicability of **MOR-3@pda@cotton fabric** as a passive sampling sorbent phase for the continuous uptake of AuNPs from water samples. Passive sampling is a popular environmental monitoring technique that relies on the passive diffusion of dissolved contaminants from water to a sorbent medium under nonequilibrium conditions.^51^ Various sorbent phases have been developed to uptake organic and inorganic compounds, yet no passive sampling phases for nanoparticle species have been reported.^52–55^ To this end, **MOR-3@pda@cotton fabric** was exposed to an aqueous medium fortified with trace amounts of PVP10@AuNPs (50 ng Au/mL). The details of the experimental procedure are described in the Supporting Information. The uptake kinetic curves of Figure 4b show that the accumulation of AuNPs exhibits a linear (integrative) phase up to 110 days, significantly higher than other passive samplers used for inorganic ions (Table S3). This attribute is critical due to the low levels of AuNPs in natural waters,^44,45,47^ which may necessitate prolonged application to concentrate an adequate amount of AuNPs. The efficiency of **MOR-3@pda@cotton fabric** for the passive sampling of AuNPs from natural waters is also reflected in the sampling rate (*R*_s_ = 2.1 mL/h), which is comparable to those obtained with commercial passive samplers for dissolved metal ions.^52–55^ SEM and PXRD measurements after **25** days of exposure show no alteration of the sorbent properties (Figure S15), which are supportive of the efficient stability of the sorbent in aqueous media for long periods. In addition, no Zr was detected by ICP-OES. Therefore, the sorbent can be useful either as an environmental monitoring tool (currently unavailable, neither commercially nor reported in the literature) or as a passive remediation tool for removing Au species from natural waters over long periods.

#### 3.3.3 Uptake of Gold from E-Waste Leachates

The utility of the **MOR-3@pda@cotton fabric** for gold recovery from electronic waste leachates was also examined using an electronic waste simulant solution^56^ containing 1500 ppm of Cu, 100 ppm of Ni, 15 ppm Al, and 10 ppm Au ions, adjusted to pH 3 with HCl (details for the preparation of the e-waste simulant are given in Supporting Information). The uptake of gold was performed under dynamic, flow-through conditions in a column cartridge packed with a **MOR-3@pda@cotton fabric** (12.56 cm^2^) (Figure 4c). The column was fitted to a vacuum filtration apparatus, and the e-waste simulant solution was percolated through the column under a vacuum at a flow rate of 2.0 mL/min. Despite the relatively fast flow rate, **MOR-3@pda@cotton fabric** removed more than 97% of Au, whereas the removal efficiencies for Cu, Ni, and Al were 7.3%, 0%, and 0%, respectively. The observed Au removal efficiency is exceptional considering the high excess of the coexisting cations (up to 464-fold excess) and anions (930, 34, and 37-fold excess for NO_3_^–^, Cl^–^, and SO_4_^2–^, respectively) compared to Au. Thus, **MOR-3@pda@cotton fabric** exhibits exceptional selectivity for Au over common ionic species in electronic waste solutions, making it promising for Au recycling applications. The selectivity of the material for Au ions is explained by the fact that the Au^3+^ ion is a soft metal ion, whereas Cu^2+^ and Ni^2+^ are borderline acids, and Al^3+^ is a hard acid.^57,58^ Thus, the material bearing soft S, N donor atoms preferably binds to the soft Au^3+^ ions. Furthermore, the transformation (reduction) of Au^3+^ to Au NPs (see below) provides an additional driving force for the selective sorption of Au^3+^ ions. Finally, we also tested the selectivity of the sorbent against other soft metal ions such as Pt and Pd under dynamic, flow-through conditions. The results revealed that the sorption efficiency for Au ions was reduced to ∼ 55% in solutions containing the same analytical concentration of all ions (Pt, Pd, Au = 10 ppm). This indicates the Pt and Pd ions compete with Au for the available adsorption sites. Batch sorption experiments with Pt and Pd (20 and 80 ppm, respectively), in the absence of Au, confirmed that MOR-3@pda@cotton fabric is capable of capturing Pt and Pd ions with removal efficiencies of 50% and 80%, respectively. However, the abundance of these elements in relation to Au in e-waste is too low,^59–61^ posing no threat to the selectivity and the efficiency of the sorbent. On the other hand, these data suggest the potential use of the sorbent for the uptake of Pt and Pd from leachates, such as those obtained from the recycling process of automobile catalytic converters, thereby expanding the potential applications of the sorbent to a broader field of precious metal recovery.

#### 3.3.4 Recovery of Au Ions and AuNPs from **MOR-3@pda@cotton fabric**-Reusability of the Sorbent

The recovery of AuNPs and Au ions from the **MOR-3@pda@cotton fabric** was examined using two elution solvents consisting of aqua regia and an equimolar mixture of NaOH/H_2_O_2_. Aqua regia was tested because it can dissolve AuNPs to AuCl_4_^–^ ions,^21,44^ enabling the recovery of Au species in their ionic form. On the other hand, strongly alkaline media and H_2_O_2_ can decompose MOFs (through oxidation of the ligand and formation of the insoluble zirconium­(IV) hydroxide precipitates) and release AuNPs without decomposing them to their ionic precursors.^21,45^ Meanwhile, recovery of Zr as hydroxide/oxide can also be achieved using NaOH/H_2_O_2_. In all experiments, 5 mL of elution solvent was used to ensure the elution volume was adequate for flame AAS aspiration.

The efficiency of aqua regia and NaOH/H_2_O_2_ was tested at different concentrations, elution times, and elution methods. As shown in Figure S16a, the dissolution efficiency declines with decreasing aqua regia acidity but remains constant for NaOH/H_2_O_2_ concentrations of 0.1 M or higher. Due to the strong acidity of the aqua regia, the elution time did not affect the extraction efficiency of AuNPs. On the other hand, 50 min of incubation in NaOH/H_2_O_2_ was necessary to completely digest the MOF and release the AuNPs (Figure S16b). The elution is optimally performed by mixing without the use of additional energy sources such as heating or sonication, which is advantageous because it reduces energy requirements (Figure S16c). Overall, NaOH-H_2_O_2_ provided, on average, 10% higher recoveries compared to aqua regia.

The efficiency of both elution solvents was examined with Au ions and AuNPs of different sizes and surface functionalization. The results show high recovery (>80–100%) for AuCl_4_^–^ ions (Figure S16d) and AuNPs (Figure S16e,f), suggesting that both aqua regia and NaOH/H_2_O_2_ can efficiently recover Au species from the MOF. The selection of the most suitable elution media can be made depending on the desired composition of the eluate (i.e., Au ions or AuNPs). However, neither elution solvent enabled the reuse of **MOR-3@pda@cotton fabric**. **MOR-3** was digested to Zr hydroxide/oxide in alkaline solutions, while the fabric deteriorated under strongly acidic and alkaline conditions. Although deterioration of the fabric was observed even when using milder acidic solutions, such as thiourea-HCl (0.01 M solution), the **MOR-3@pda@cotton fabric** sorbent offers exceptional sorption capacity, allowing for its extensive use before reaching saturation. Additionally, the straightforward preparation of the material, involving an easily synthesized organic linker, an inexpensive bulk support (i.e., cotton fabric), and a simple experimental setup with moderate reaction temperatures, mitigates this limitation. Nonetheless, intending to address the reusability of the sorbent we prepared a MOR-3-calcium alginate (CA) composite in the form of beads (see Supporting Information for synthesis), which were then used as a stationary phase in a column for the sorption of Au ions (initial concentration of 20 ppm, pH = 3.74) under continuous flow conditions. Five adsorption and desorption cycles were performed, and regeneration was achieved by washing the column with an acidic thiourea solution (Figure 4d). The beads became darker upon contact with the Au ion solution, indicating the reduction of Au ions to AuNPs (see below) (Figure 4e). The yellow color of the beads was restored upon treatment of the Au-loaded material with the acidic thiourea solution (Figure 4e). The results revealed adsorption and recovery values of more than 85 and 87%, respectively, demonstrating the excellent reusability of the material (Figure 4d). PXRD data indicated that sorbent retained its crystallinity after the sorption/desorption studies (Figure S17). Notably, the reusability of the material was shown under continuous flow conditions, eliminating the need for sorbent isolation via filtration or centrifugation between runs, which are time-consuming processes and unsuitable for large-scale applications. Furthermore, by avoiding filtration or centrifugation, the removal or precipitation of AuNPs, which may lead to overestimated sorption efficiency, is prevented.^62^ Thus, the observed removal capacities can be attributed exclusively to sorption.

### 3.4 Comparison with Known Sorbents

Table S4 summarizes the sorption properties of some of the most effective MOFs and composites as sorbents for Au ionic species.^2^ From these data, it can be inferred that several materials are available for capturing Au ions from aqueous media, demonstrating high sorption capacities and fast sorption kinetics. However, most sorbents are only efficient under strongly acidic conditions (pH 1–3).^63,64^ In contrast, the sorbent presented in this work can capture Au ions in a wide pH range (3–8) and shows one of the highest sorption capacities (∼900 mg/g) among MOFs and composites.^2,63,65^

Furthermore, most sorbents have been tested for removing and recovering Au ionic species, ignoring AuNPs, which are widely used in many technological and medical applications. Only a limited number of studies have reported the uptake of AuNPs (Table S4), typically requiring prolonged incubation times to accomplish the extraction.^66,67^ Moreover, the recovery of AuNPs from the known sorbents was low or lacked selectivity.^26,66,67^ Additionally, most reported sorbents have been mainly prepared and tested in powder form, which is inconvenient for realistic applications.^2^

The material described in this work provides a rare example that demonstrates the capability to capture and recover Au ions and Au nanoparticles simultaneously. Furthermore, it is based on a MOF immobilized on cotton fabrics, i.e., an inexpensive bulk support, that can be prepared via a straightforward route and can be directly employed and recovered from natural waters and wastewater. Not the least, compared to our previous work describing the sorption of Au ions and AuNPs on a Zr^4+^-mercaptosuccinate MOF (MOF-SH),^21^ the sorbent described here exhibits improved air stability (due to the lack of easily oxidizable thiol units) and significantly higher sorption capacities for Au ions (889 mg Au/g **MOR-3** vs 144 mg Au/g **MOF-SH)** and AuNPs (35.4 mg Au/g **MOR-3** vs 28.7 mg Au/g **MOF-SH** for AuNPs) (both compared in their powder form).

### 3.5 Postsynthetic Characterization of Au-loaded Materials-Mechanism of Sorption

PXRD (Figure 5a) and Le Bail analysis for the Au-loaded materials (Figure S18) proved that the structure and crystallinity of the MOF are preserved after the sorption process. After the sorption of AuNPs and Au ions, the yellow color of the **MOR-3** powder turned to pale brown and purple, respectively (Figure S19). These color transitions were not evident in the **MOR-3@pda@cotton fabric** due to the black coloration of the cotton fabric after treatment with PDA (Figure S19a). The solid-state UV–vis spectra of Au ions and AuNPs-loaded materials, depicted in Figure 5b, exhibit absorption bands at 545 and 525 nm, characteristic of gold nanoparticles. This indicates that Au ions are reduced to Au nanoparticles during sorption. Furthermore, backscattered electron (BSE)-SEM images and energy-dispersive spectroscopy (EDS) analysis revealed the distribution of AuNPs as bright spots in both Au ion and AuNPs-loaded materials (Figures 5c and S20).

**Figure 5:**
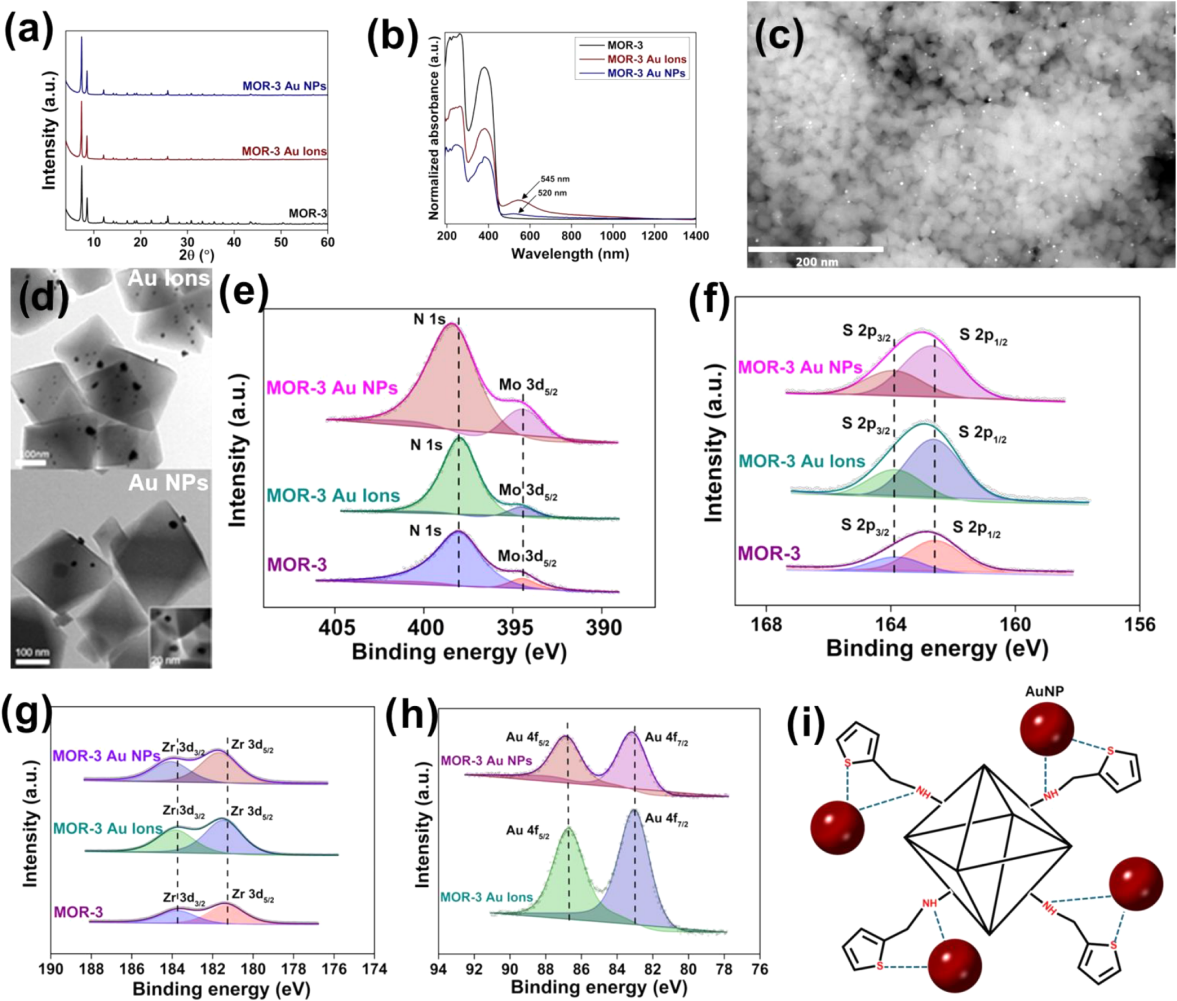
(a) Comparative PXRD patterns and (b) Comparative UV–vis spectra of pristine MOR-3, Au ions, and AuNPs MOR-3-loaded materials. (c) FE-SEM of Au ions-loaded material and (d) TEM images of Au ions and AuNPs MOR-3-loaded materials. The inset shows a high-magnification TEM image of the AuNPs-loaded sample. (e) High-resolution N 1s core-level photoelectron spectra of pristine MOR-3, Au ions, and AuNPs-loaded materials. Mo peak is due to the holder. (f) High-resolution S 2p core-level photoelectron spectra of pristine MOR-3, Au ions, and AuNPs-loaded materials. (g) High-resolution Zr 3d core-level photoelectron spectra of pristine MOR-3, Au ions, and AuNPs-loaded materials. (h) High-resolution Au 4f core-level photoelectron spectra of Au ions and AuNPs-loaded materials. (i)­Representation of the Au sorption mechanism by MOR-3.

SEM data provide additional evidence for the reduction of Au ions to AuNPs during the continuous flow sorption studies with the MOR-3-CA composite (Figure S21). Furthermore, TEM images demonstrated the distribution of AuNPs, as dark spots, on the surface of **MOR-3** particles, with sizes of 11.8 ± 5.1 and 15.4 ± 2.1 nm for the materials treated with Au ions and AuNPs, respectively (Figure 5d). The BET surface area of the Au-ions-loaded material (350 m^2^/g) was reduced by ∼48% compared to that of the pristine MOF (Figure S22). This decrease in the BET surface area may be attributed to the partial closure of pores by the sorbed Au ions and Au nanoparticles. Likely, the sorption of Au ions begins with forming Au–S/N bonds, resulting in the development of a first layer of Au atoms that partially blocks the pores of the MOF structure. This is followed by reducing Au ions to neutral Au atoms and the subsequent growth of AuNPs. In contrast, the BET surface area of the AuNPs-loaded material (687 m^2^/g) is slightly higher than that of the pristine MOF (Figure S23). The substantial size of AuNPs hindered their efficient penetration into the pores and surface coverage of MOF particles, resulting in the formation of AuNPs assemblies on the MOF surface. This led to the creation of additional porous agglomerates with interparticle voids. This may be the reason for the slight increase in the BET surface area of the AuNPs-loaded material. Similar observations have also been made with the Zr^4+^-mercaptosuccinate MOF.^21^ Pore size distribution analysis for **MOR-3** loaded with Au ions and AuNPs indicated no alterations in pore widths for both materials, which are identical to that of the pristine MOF (∼5.2 Å), (Figure S10e,f). The identical pore sizes of pristine and Au-loaded materials are consistent with the sorption of AuNPs on the external surface of MOF particles. X-ray photoelectron spectroscopy (XPS) was also employed to gain insight into the interactions between AuNPs and the MOF framework and identify the oxidation state of Au in the loaded materials (Τable S5). The results revealed a positive shift (by 0.1–0.4 eV) for the S 2p_3/2_ and 2p_1/2_ lines, the N 1s line, as well as the Zr 3d_5/2_ and 3d_3/2_ lines in the AuNPs-loaded sample compared to the corresponding values for the pristine material (Figure 5e–g). Furthermore, the Au 4f_7/2_ and 4f_5/2_ core-level spectra of the AuNPs-loaded material indicate binding energies of 83.1 and 86.9 eV, respectively, which are somewhat lower than the typical values for metallic Au, indicating the presence of a partial negative charge on the Au atoms (Figure 5h).

The above results demonstrate an electron transfer from the MOF framework to AuNPs, with N and S atoms likely coordinating to Au metal centers (Figure 5i). Similar results were obtained for the MOF treated with Au ions. However, the shift in binding energies is less pronounced in this case (Figure 5e–h). Specifically, a limited shift of 0.1 eV was noted for the S 2p_3/2_ and 2p_1/2_ lines as well as the Zr 3d_5/2_ and 3d_3/2_ lines of Au ion-loaded material, whereas no discernible change was observed for the N 1s line. In addition, the Au 4f_7/2_ and 4f_5/2_ signals indicate binding energies of 83 and 86.7 eV, respectively, further corroborating the partially negative charge on the Au atoms. These data are consistent with an electron transfer from the MOF to the Au species. The extent of electron transfer is anticipated to diminish in **MOR-3** treated with Au ions compared to **MOR-3** loaded with AuNPs, owing to the smaller size of Au species and concomitant weaker electronic interactions between Au ions and the **MOR-3** surface. As a result, less pronounced changes were observed in the XPS spectra of **MOR-3** loaded with Au ions.^68^ Finally, a question remains regarding the cause of the reduction of Au ions to AuNPs in **MOR-3**. An examination of the XPS data reveals no alterations in the N 1s line, suggesting that the amine groups of MOF are not implicated in the reduction process. A potential reducing agent is dimethylformamide (DMF), present in relatively small quantities in **MOR-3**. Another candidate for reducing Au ions to AuNPs is triethylamine (Et_3_N), which is detected in **MOR-3** due to its pretreatment with a methanolic Et_3_N solution (Figure S24).

## 4 Conclusions

A Zr­(IV) MOF functionalized with amino and thiophene groups (**MOR-3**) and a microporous defective structure was isolated and structurally characterized for the first time via MicroED. The MOF was deposited in situ on cotton fabric, forming a MOF-decorated fabric material (**MOR-3@pda@cotton fabric**), thoroughly examined as a sorbent for capturing Au ions and Au nanoparticles. The material demonstrated efficient sorption kinetics, remarkably high sorption capacities, significant recoveries, and the capability to uptake Au ions and AuNPs from natural waters, independently of AuNP coating and size or the complexity of the water matrix. The excellent Au sorption properties of the MOF sorbent arise from the amino and thiophene groups firmly bound to Au species, as demonstrated from various experimental data. For applications related to gold recovery, **MOR-3@pda@cotton fabric** was investigated for the selective uptake of Au species from electronic waste simulants with very satisfactory results. Moreover, the sorbent exhibited high performance as a passive sampler for monitoring AuNPs in environmental waters. Although the MOF-fabric sorbent is not reusable due to the deterioration of cotton under acidic or basic conditions, it offers a high sorption capacity, allowing for its extensive use before saturation. Still, we managed to address the reusability issue by preparing a MOF-calcium alginate composite. The latter demonstrated an exceptional capability for adsorbing and desorbing Au species under continuous flow conditions over five consecutive cycles. Overall, the results emphasize the potential of MOFs bearing soft bases as promising sorbents for gold recovery. Furthermore, immobilizing such MOFs on bulk supports represents a step toward real applications. Future work will involve the development of MOF-based sorbents with hierarchical porous frameworks to increase sorption capacities while retaining strong noble metal-MOF interactions.

## Supplementary Material



## Notes

Additional experimental details, materials, and methods, Crystallographic Information File (CIF) for **MOR-3**, PXRD patterns, TGA, ED, and FE-SEM images, ^1^H NMR, EDS, N_2_ and CO_2_ adsorption isotherms, pore distribution diagrams, kinetics and isotherm data, tables with comparisons of Au sorbents and binding energies (PDF)
