# Supplementary material for: An Amino-Thiophene Functionalized Metal–Organic Framework on Fabric for Selective Extraction, Recovery, and Passive Sampling of Gold Ions and Nanoparticles
Source: Chem Mater. 2025 Sep 2;37(18):7147–58. doi: 10.1021/acs.chemmater.5c01238 (PMC12462624; doi:10.1021/acs.chemmater.5c01238)
Supplement: Supplementary file 1 [file cm5c01238_si_001.pdf]

Supporting Information

An Amino-Thiophene Functionalized Metal-Organic Framework on Fabric for Selective Extraction, Recovery, and Passive Sampling of Gold Ions and Nanoparticles

Vasiliki Gouma,[a,] Eleni C. Makri,[a] Evangelos K. Andreou,[b] Emilia Buchsteiner,[c] Gerasimos S. Armatas,[d] Manolis J. Manos[a*] and Dimosthenis L. Giokas[a*]

[a] Department of Chemistry, University of Ioannina, GR-45110 Ioannina, Greece

[b] University of St Andrews, School of Chemistry, St Andrews, KY16 9AL, UK

[c] Rigaku Europe SE Hugenottenallee 167, 63263 Neu-Isenburg, Germany

[d] Department of Materials Science and Technology, University of Crete, GR-70013 Heraklion, Greece

Table of contents

Experimental section S2
  Materials S2
  Instrumentation S2
Syntheses S4
  Synthesis of MOR-3 .... S4
  Synthesis of AuNPs S4
  Synthesis of MOR-3@CA beads S5
Batch sorption studies S5
  Preparation of electronic waste simulant solution S6
  MOR-3@pda@cotton fabric as passive sampling receiving phase S6
Characterization techniques S7
  Details of the structure refinement with MicroED data S7
References S26

Experimental section

Materials

Thiophene-2-carboxaldehyde, 98+% was from Thermo Fisher Scientific (Kandel, Germany). Zirconium chloride (99.5%+, metal basis) was obtained from Alfa-Aesar (Kandel, Germany). 2-aminoterephthalic 99% (NH_2-BDC) was from Acros Organics (Geel, Belgium). Hydrogen tetrachloroaurate trihydrate (≥99.9% trace metals basis), sodium borohydride, tri-sodium citrate, polyvinyl pyrrolidone (PVP_{10}, MW 10,000), cetyltrimethylammonium bromide (CTAB), tris(hydroxymethyl) aminomethane (tris-base) 99.9%, L-Cysteine 96%, L- ascorbic acid and glacial acetic acid ≥99.7% were obtained from Sigma-Aldrich (Steinheim, Germany). 3-hydroxytyraminium chloride ≥99.0%, hydrogen peroxide solution 30%, N, N-Dimethylformamide (for synthesis) and acetone were procured from Merck (Darmstadt, Germany). Glycine (analytical grade) was obtained from Serva Fein Biochemica (Heidelberg, New York). HPLC-grade methanol was retrieved from Fisher Scientific (Loughborough, UK). Hydrochloric acid (puriss. p.a.) and diethyl ether were obtained from Honeywell Fluka (Seelze, Germany). Nitric acid 65% G.R. was obtained from Lachner (Neratovice, Czech Republic). Sodium hydroxide was from Mallinckrodt (Dublin, Ireland).

Instrumentation

A Shimadzu AA-6800 (Shimadzu Corp., Kyoto, Japan) atomic absorption spectrophotometer (FAAS) with a self-reversal hollow cathode lamp (Heraeus, Hanau, Germany) operating at 10 mA was used for the measurements of gold ions and AuNPs, which were made at 242.8 nm. Instrumental calibration was performed according to the manufacturer's specifications using standard solutions of gold ions. To estimate the nanoparticles' size, spectrophotometric measurements were performed in a Jenway 6405 UV/Vis spectrophotometer (Essex, UK) with matched quartz cells of 1 cm path length.

The concentration of Zr^{4+} in aqueous samples was determined with ICP OES (Shimadzu ICPE-9800) equipped with a semiconductor CCD detector. The readouts were recorded at an exposure time of 30 s in the axial view mode. The plasma torch was operated with an RF power of 1.2 kW at 27 MHz frequency, coolant argon flow

rate of 10 L min-1, auxiliary argon flow rate of 0.6 L min^{-1}, and carrier flow rate of 1.0 mL min^{-1}. The analytical signals were recorded at 339.198 nm.

Powder XRD (PXRD) diffraction patterns were recorded on a Bruker D2 Phaser X-ray diffractometer (CuKα radiation, wavelength = 1.54184 Å). Scanning electron microscopy (SEM) measurements were performed at the Microscopy Unit of the Chemistry Department with a Phenom Pharos G2 Desktop FEG-SEM (Thermo Fisher Scientific) on Cr sputtered specimens (Q150T ES Plus automatic sputter coater, Quorum Technologies Ltd.) IR spectra were recorded on a Perkin Elmer Spectrum Two attenuated total reflectance-IR (ATR-IR) spectrometer. Thermogravimetric data (TGA plots) were collected from a Mettler-Toledo TGA/DSC1 instrument under an O_2 flow of 50 mL/min. The UV-Vis diffuse reflectance spectra of solid samples were collected using a Shimadzu UV-2600i spectrophotometer equipped with an ISR-2600Plus integrating sphere in the 190-1400 cm^{-1} wavelength range. $BaSO_4$ powder was used as a reference and base material on which the powder sample was coated. The reflectance data were converted to absorption data using the Kubelka–Munk equation. ^1H NMR spectra were measured with a Bruker 250 MHz spectrometer. N_2 adsorption-desorption isotherms and CO_2 physisorption measurements were recorded at 77 and 273 K, respectively, on a Quantachrome Nova 3200*e* sorption analyzer. Before analysis, all samples underwent degassing at 80 ^oC under vacuum ($<10^{-5}$ Torr) for 12 h. The specific surface areas were determined by applying the Brunauer-Emmett-Teller (BET) method to the absorption branch of isotherms within the 0.04–0.23 relative pressure (P/P_o) range. The corresponding pore-size distribution plots were obtained by fitting the CO_2 adsorption data of the isotherms to the nonlocal density functional theory (NLDFT) model. Transmission electron microscopy (TEM) images were obtained with a JEOL JEM-2100 electron microscope (LaB6 filament) operating at 200 kV. The samples were prepared by suspending fine powders in ethanol using sonication and then drop-casting on a carbon-coated Formvar Cu grid. XPS measurements were performed on a SPECS spectrometer using a Phoibos 100 1D-DLD electron analyzer and an Al Kα radiation as the energy source (1486.6 eV). Binding energy values were corrected for charging by assigning a bending energy of 284.8 eV to the C 1s signal of adventitious carbon. Zr, Au, N, and S XPS analysis was performed based on the Zr 3d, Au 4f, N 1s, and S 2p signals. Three-dimensional electron diffraction (3D ED) was employed to determine the structure of **MOR-3** microcrystallites.[1,2] Diffraction data were collected using an

XtaLAB Synergy-ED electron diffractometer (Rigaku and JEOL collaboration). The sample was prepared by gently grinding it between two glass plates and depositing it onto a continuous carbon copper grid. It was then transferred to a Gatan Elsa specimen holder via a cryo-chamber (liquid nitrogen chamber), and the crystallites were measured at 175 K with a wavelength of 0.0251 Å. Four measurements, each lasting from four to eight minutes, were conducted. Structure solution and refinement were performed with the ShelX and Olex2 software suites using the merged diffraction data of the best two data sets.[3,4]

CCDC 2451528 contains the supplementary crystallographic data for this paper.

Syntheses

Synthesis of MOR-3

$ZrCl_4$ (0.257 mmol) and H_2TATP (0.36 mmol) were dissolved in a mixture of dimethylformamide (DMF, 8 mL) and acetic acid (1.2 mL) in a 20 mL glass vial. The mixture was sonicated for 5 minutes and then left at 120 ^oC inside an isothermal oven for 4 hours. The final mixture was centrifuged, and the isolated solid product (yellowish fine powder) was washed with DMF, water, and acetone, and then it was left to dry for 1 day at 80 ^oC. Yield: 0.075g.

Synthesis of AuNPs

Citrate-capped AuNPs (CA@AuNPs) of variable average size distribution were synthesized by reduction of $AuCl_4^-$ from $NaBH_4$ in the presence of tri-sodium citrate as stabilizer or by using tri-sodium citrate both as a reducing agent and as a stabilizer, using standard procedures reported in the literature.[5–7]

Using the as-synthesized CA@AuNPs, various AuNPs with different coatings were prepared according to previous methods.[8,9] PVP10-coated AuNPs were prepared by mixing 10 mL of an aqueous CA@AuNPs suspension with 1.1 mL of PVP10 (0.02 mM in distilled water), stirred for 20h. Cysteine-coated AuNPs (Cys@AuNPs) were prepared by mixing 10 mL of a CA@AuNPs suspension with 200μL cysteine solution (1.0 mM), stirred for 10 min, and aged 24 h in the dark. Glycine-capped AuNPs (Gly@AuNPS) were prepared by adding 200μL of glycine (1 mM) to 10 mL of an aqueous CA@AuNPs suspension under continuous stirring for 20 min at 1000 rpm. Synthesis of CTAB-stabilized AuNPs (CYAB@AuNPs) was performed according to

the seeding growth method.[7] The average size distribution and the molar concentration of the synthesized AuNPs were calculated from their UV-Vis spectra.[10] Specifically, the average size distribution in each AuNP suspension was first calculated by the ratio of the absorbance of AuNPs at the surface plasmon resonance peak to the absorbance at 450 nm. Based on the estimated size distribution, the concentration of AuNPs was calculated by dividing the absorbance at 450 nm by the molar decadic extinction coefficient at λ=450 nm.[10]

Synthesis of MOR-3@CA beads

0.120g of alginic acid was dissolved in 20mL of distilled water via sonication and vigorous stirring at 40 °C until total dissolution. Then 1g of MOR-3 was dispersed, and the mixture was stirred for 2 hours. The resulting suspension was dropped through a Pasteur pipette into a 2% (w/v) $CaCl_2$ solution, and the formed MOR-3 beads were left for 1h in a gelatinization bath. Isolation was achieved through filtration in a Buchner funnel, and the excess amount of unbound calcium was removed through excess washing with water. Calcium alginate beads were dried overnight at 60 °C. (Yield:1.03g beads)

Batch sorption studies

Batch sorption experiments were conducted at room temperature with a volume-to-mass (MOR-3) ratio (V:m) of 6000 mL/g. Sorption kinetics of AuNPs were studied using 50 mL aqueous solutions and a semicircular (12.56 cm^2) **MOR-3@pda@cotton fabric** at pH 3, using 5.6 nM PVP@Au NPs (4nm) (3 mg/L as $AuCl_4^-$ ions), while the sorption kinetics of $AuCl_4^-$ ions was studied at pH 6, also at 3 mg/L of $AuCl_4^-$ ions. The solutions were agitated at different reaction times ranging from 1 to 1440 min in an orbital shaker at 150 rpm. At specific intervals, atomic absorption spectroscopy analyzed the aqueous supernatant's content in AuNPs or $AuCl_4^-$ ions (expressed as mg/L $AuCl_4^-$ ions).

The uptake of AuNPs by **MOR-3@pda@cotton fabric** was investigated at pH 3 by determining the sorption efficiency of PVP@AuNPs (5 nm) (0.84-134.4 nM PVP@AuNPs containing 0.5–80 mg L^{-1} Au ions, respectively) after 3 h of mixing in an orbital shaker at 150 rpm. The sorption of $AuCl_4^-$ ions was studied at pH 6 in the

presence of 0.5–500 mg L^{-1} $AuCl_4^-$ ions after 1 h of orbital mixing at 150 rpm. All experiments were run in triplicate, and the results were averaged.

The above batch sorption experiments were also performed using 8.0 mg of **MOR-3** powder, previously treated with a methanolic triethylamine solution (100mg of **MOR-3** in a mixture of 4 mL methanol and 0.1 mL triethylamine), to compare the sorption properties of **MOR-3** with those of **MOR-3@pda@cotton fabric**.

Preparation of electronic waste simulant solution

The e-waste stimulant solution was prepared from $NiSO_4 \times 6H_2O$, $Cu(NO_3)_2 \times 3H_2O$, $AlCl_3$ and $AuCl_4 \times 3H_2O$ salts. The final composition of the mixture was 1500 ppm Cu^{2+}, 100 ppm Ni^{2+}, 15 ppm Al^{3+}, 10 ppm Au ions, 165 ppm SO_4^{2-}, 3000 ppm NO_3^- and 70 ppm Cl^-. The pH of the mixture was adjusted to 3.0±0.5 using HCl.

MOR-3@pda@cotton fabric as passive sampling receiving phase

The calibration of **MOR-3@pda@cotton fabrics** as passive samplers was performed by static renewal exposure experiments.[11] Eight semi-circular fabrics of 12.56cm^2 surface area each were fixed on a metallic holder (stainless steel mesh of 1mm wire thickness and 6mm mesh pore size) and immersed in a 3 L aqueous sample solution fortified with 0.094 nM PVP@AuNPs (50μg L^{-1} Au). The sample was mixed by stirring at 300 rev/min, at 20°C, and renewed daily to ensure constant exposure. The mixing speed was decided to correspond to a horizontal (linear) flow velocity of 1.72 m/s that is typical of a moderately fast river and calculated from the formulae $u = \frac{2\pi r}{60} \times N$ where u (m/s) is the linear flow velocity, r is the length of the magnetic rod (in meters) and N is the angular velocity (in rpm). To investigate the accumulation kinetics of AuNPs in the **MOR-3@pda@cotton fabric** phase, one fabric was removed from the vial weekly, extracted with $NaOH/H_2O_2$, and analyzed for its content in AuNPs. The calibration of the passive sampler was performed for as long as the

uptake of AuNPs over time was linear (110 consecutive days). The data were used to calculate the sampling rate (R_s, L d^{-1}), which is the volume of water extracted per unit of time, and is reprsented by the formula: $Rs = \frac{M}{Cw \times t}$

where M(μg) is the amount of a chemical accumulated in the sampler, C_w (μg L^{-1}) is the concentration of AuNPs in water and t (days) is the deployment time during the linear uptake phase. The calculation of R_s was performed in the linear uptake phase of the uptake profile by taking the slope of M/C_w versus deployment time. Control experiments (water samples containing the fabric samplers but not PVP@AuNPs, and water samples containing PVP@AuNPs but not fabric samplers) were deployed to account for contamination of the samplers or loss of analytes, not attributed to PVP@AuNPs, respectively. The results showed no contamination of the samplers and trivial loss of PVP@AuNPs (<3.5%); therefore, correction of the calculated concentrations was not deemed necessary.

Characterization techniques

Details of the structure refinement with MicroED data

As a result of the space group symmetry (I4/m), there are two crystallographically unique dicarboxylate ligands with a ratio of 2:1. For a non-defective framework, there will be four ligands of one type and two ligands for the second type. To identify if this is an ideal or defective framework, we performed refinement for the occupancies of all C atoms, keeping the same occupancy for the C atoms of the same type and ligand (e.g., the phenyl carbons of one of the crystallographically unique ligands were forced to have the same occupancy). The occupancies for the oxygen atoms of the ligands were retained full, as these atoms represent all the possible types of oxygen (i.e., carboxylate, hydroxy, water, acetate O) ligated to Zr^{4+}. Note that C atoms bound to carboxylic groups could be phenyl or acetate atoms (as NMR indicates the presence of acetate groups). Therefore, only the phenyl C atoms not connected to carboxylate ligands exclusively belong to the dicarboxylate linkers. Determining the number of these atoms, we can thus determine the number of dicarboxylate ligands (6-x) and that of missing linkers (x). The refinement indicated an occupancy of 0.78548 for the phenyl C atoms (not connected to carboxylate groups) of the four symmetry-related ligands. Thus, these

C atoms are 16 x 0.78548 ~12.57. In addition, the occupancy for the phenyl C atoms (not connected to carboxylate groups) of the two symmetry-related ligands was 0.76499; thus, these C atoms are 8 x 0.76499 ~6.12. Hence, the total number of phenyl carbon atoms not connected to carboxylate groups is 18.69. These carbon atoms are equal to 4×(6-x). Thus, 4×(6-x)=18.69 or x~1.33. Therefore, the number of dicarboxylate linkers is 6-x=4.67; thus, the number of all C atoms of the linkers is 4.67×8=37.36. Subtracting this number from the total C atoms (41.04), there is an excess of 3.68 C atoms. These atoms belong to acetate groups. The number of acetate groups is thus 3.68/2= 1.84, not far from that calculated based on ^{1}H NMR (2.02).

Based on ^{1}H NMR of the material treated at 150 ^{o}C (to remove various solvents, not bound acetic acid, etc.), there are $TATP^{2-}$ and NH_2BDC^{2-} ligands in a ratio of 1:0.16. Based on this ratio and the total number of linkers (4.67) determined from the ED data, there are 4.03 and 0.64 moles of $TATP^{2-}$ and NH_2BDC^{2-} ligands per formula unit of the MOF, respectively. Furthermore, the ^{1}H NMR spectrum indicated the presence of acetate and DMF. The formula of a defective Zr^{4+} MOF with a monoanionic modulator, such as an acetate, can be written as $Zr_6O_4(OH)_{4+2x-y}(L)_{6-x}(modulator)_y(H_2O)_{2x-y}$ (1), considering that missing linker sites not filled with modulator will be occupied by OH^- and H_2O[12]. However, at 150 °C, no lattice and coordinated water molecules are present. Based on the ratio of integrals of the protons of the $TATP^{2-}$ ligand to those of acetate and DMF (both non-hydrolyzed and hydrolyzed DMF), the formula of the MOF treated at 150 ^{o}C is determined as

$Zr_6O_4(OH)_{4.64}(TATP)_{4.03}(NH_2BDC)_{0.64}(CH_3COO)_{2.02}(DMF)_{0.77}$.

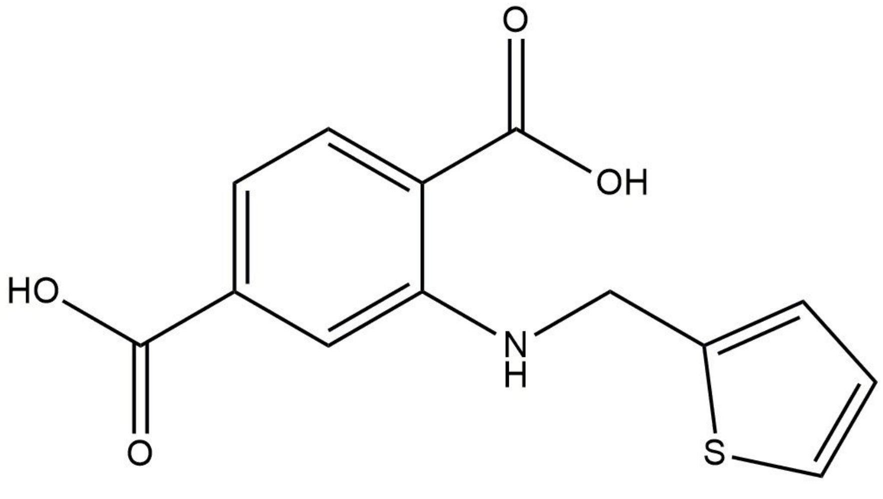

Scheme 1: Chemical structure of ligand 2-((thiophene-2-ylmethyl) amino) terephthalic acid.

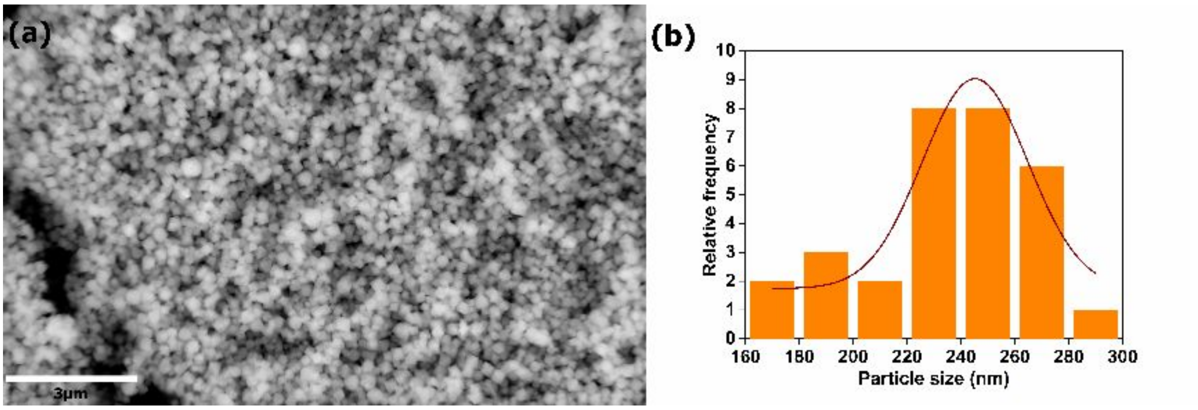

Figure S1. (a) FE-SEM image of MOR-3. (b) Particle size distribution histogram of MOR-3.

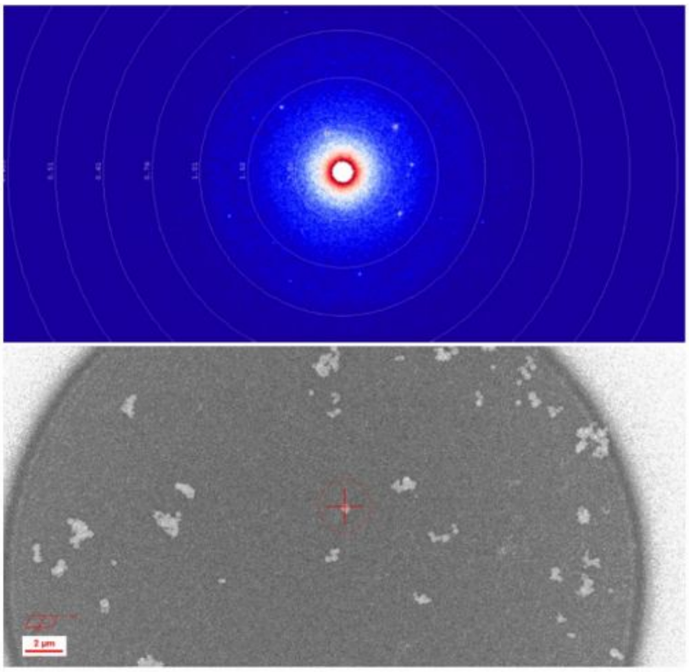

Figure S2. Diffraction image and grain snapshot of MOR-3 MicroED measurement

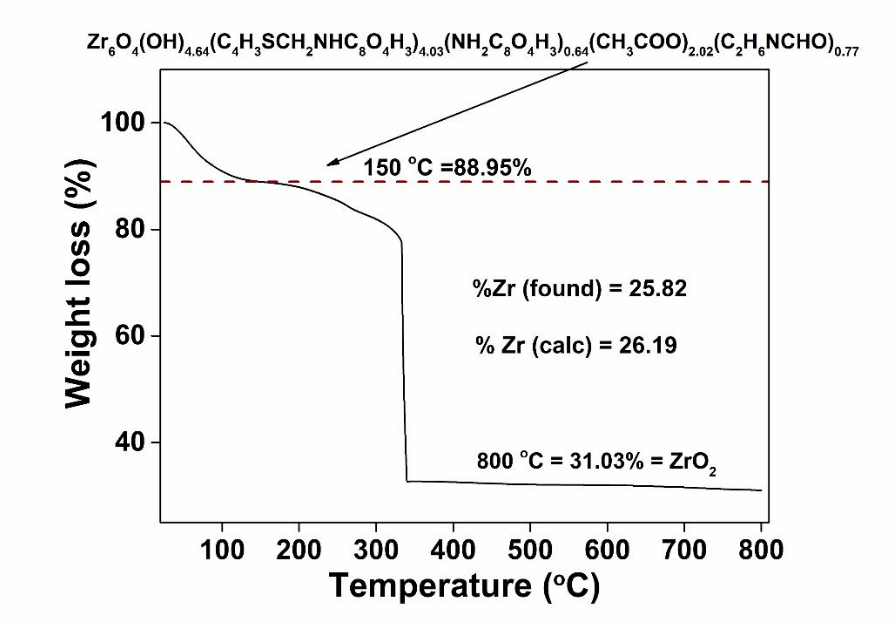

Figure S3. The TGA data for MOR-3 **were** measured under O_2 flow. The initial weight loss step, occurring from room temperature to ~150 °C, is attributed to the release of water (lattice and coordinated molecules) and some DMF molecules. Subsequent weight losses (till 800 °C) are assigned to removing linkers and residual carbon. The final residue is ZrO_2. The %Zr content calculated for the water-free material [$Zr_6O_4(OH)_{4.64}(C_4H_3SCH_2NHC_8O_4H_3)_{4.03}(NH_2C_8O_4H_3)_{0.64}(CH_3COO)_{2.02}(C_2H_6NCHO)_{0.77}$] is 26.19%, which is close to that experimentally found based on ZrO_2 (% Zr (experimental) = 25.82 %). This formula indicates a defective UiO-type Zr^{4+} MOF, with 1.33 missing linkers. DMF and acetate molecules were estimated using ^{1}H-NMR spectroscopy.

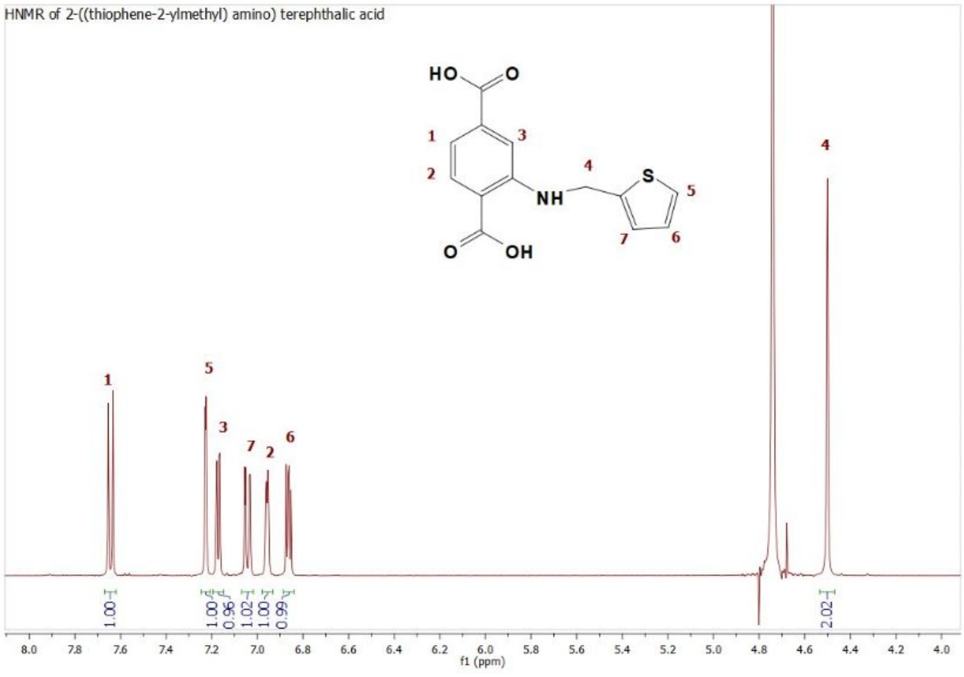

Figure S4. The ^{1}HNMR spectrum of 2-((thiophene-2-ylmethyl) amino) terephthalic acid in D_2O/NaOH solution.

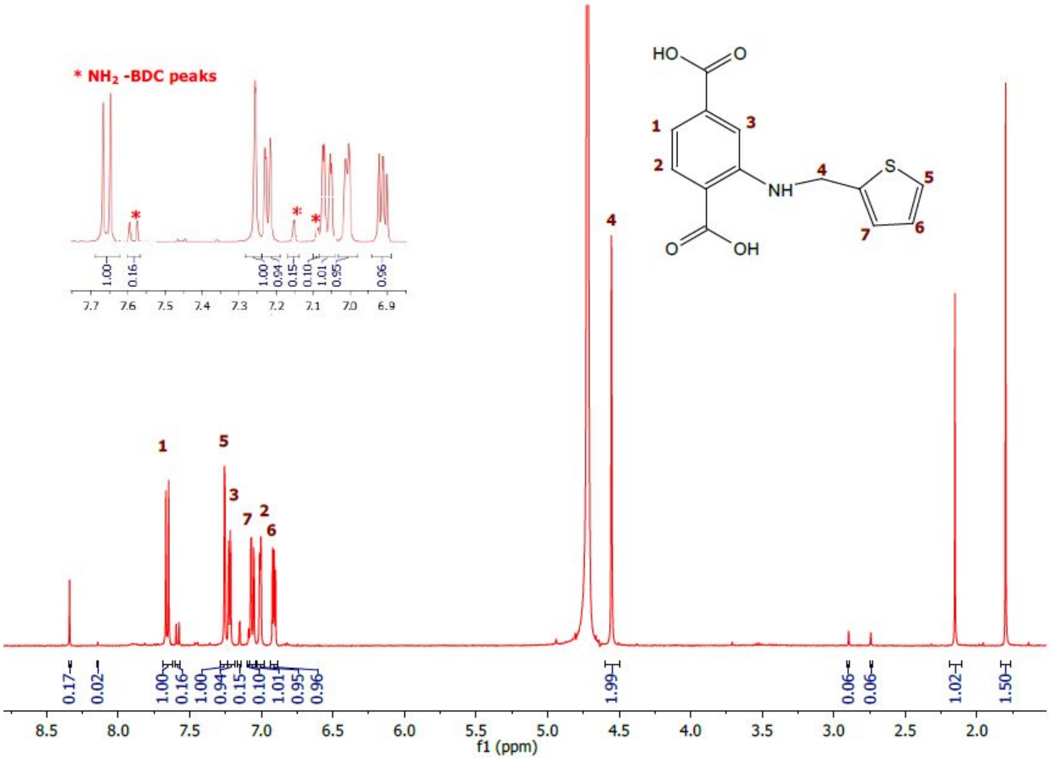

Figure S5. The ^{1}HNMR spectrum of MOR-3 after digestion in D_2O/NaOH solution. Before the ^{1}H NMR measurement, MOR-3 was preheated at 150 °C inside the TGA apparatus, so that we could relate the results from TGA with those from ^{1}H NMR studies. Labeled peaks are attributed to ligand protons of the $TATP^{2-}$ ligand. In the inset graph, peaks indicated with * belong to the NH_2BDC^{2-} ligand resulting from the decomposition of $TATP^{2-}$ ligand during the MOF synthesis. Furthermore, DMF peaks are detected at 2.75, 2.85, and 8.1 ppm. Due to the hydrolysis of DMF, formic acid and dimethylamine peaks are shown at 8.3 and 2.1 ppm, respectively. Finally, the acetate hydrogen atoms are found at 1.8 ppm.

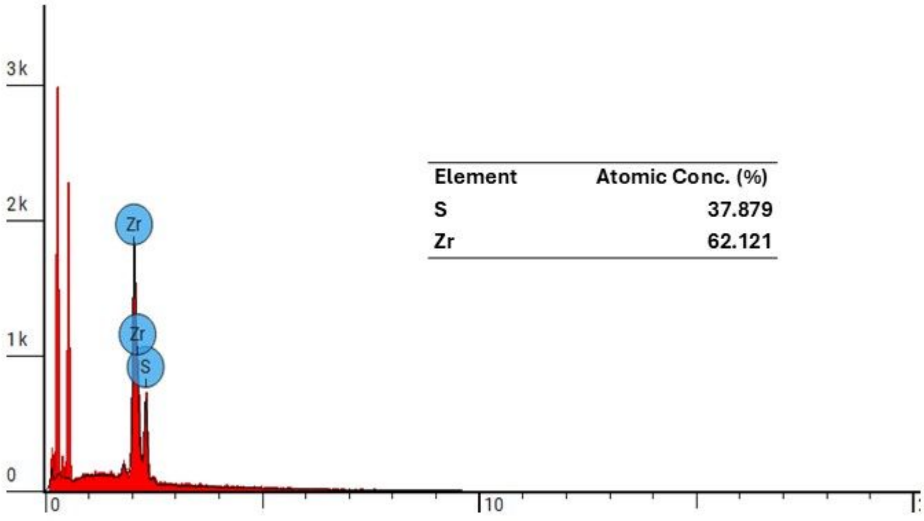

Element	Atomic Conc. (%)
S	37.879
Zr	62.121

Figure S6. EDS analysis of MOR-3.

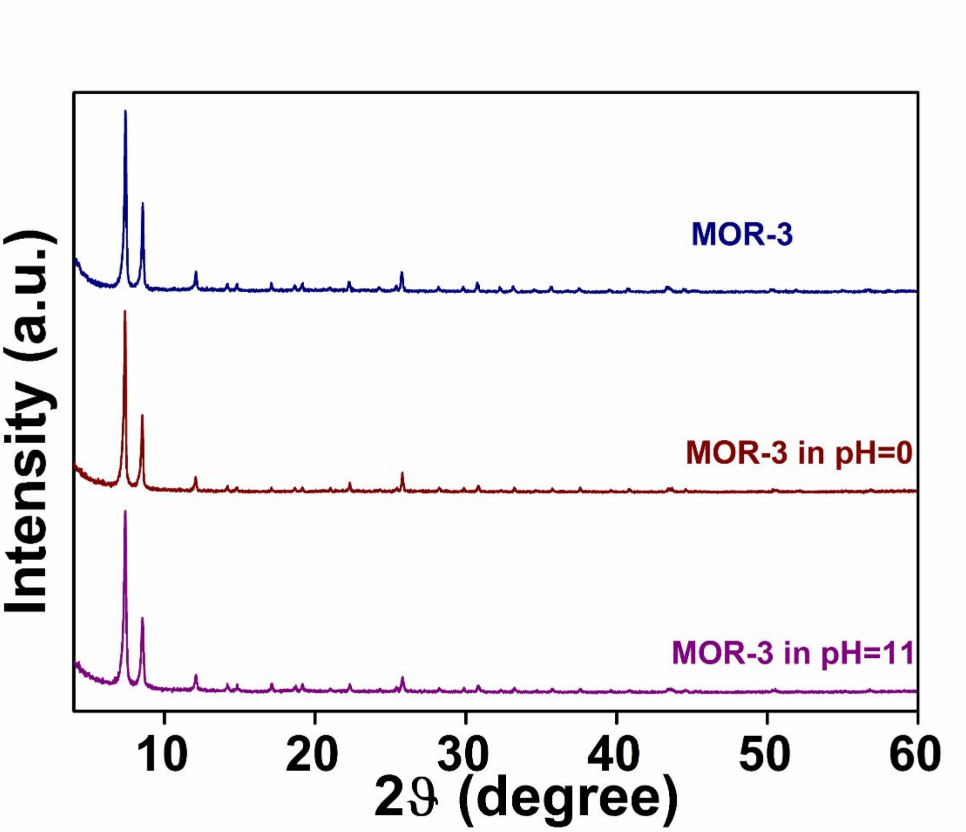

Figure S7. Stability study of MOR-3. Comparative PXRD patterns in various pH values.

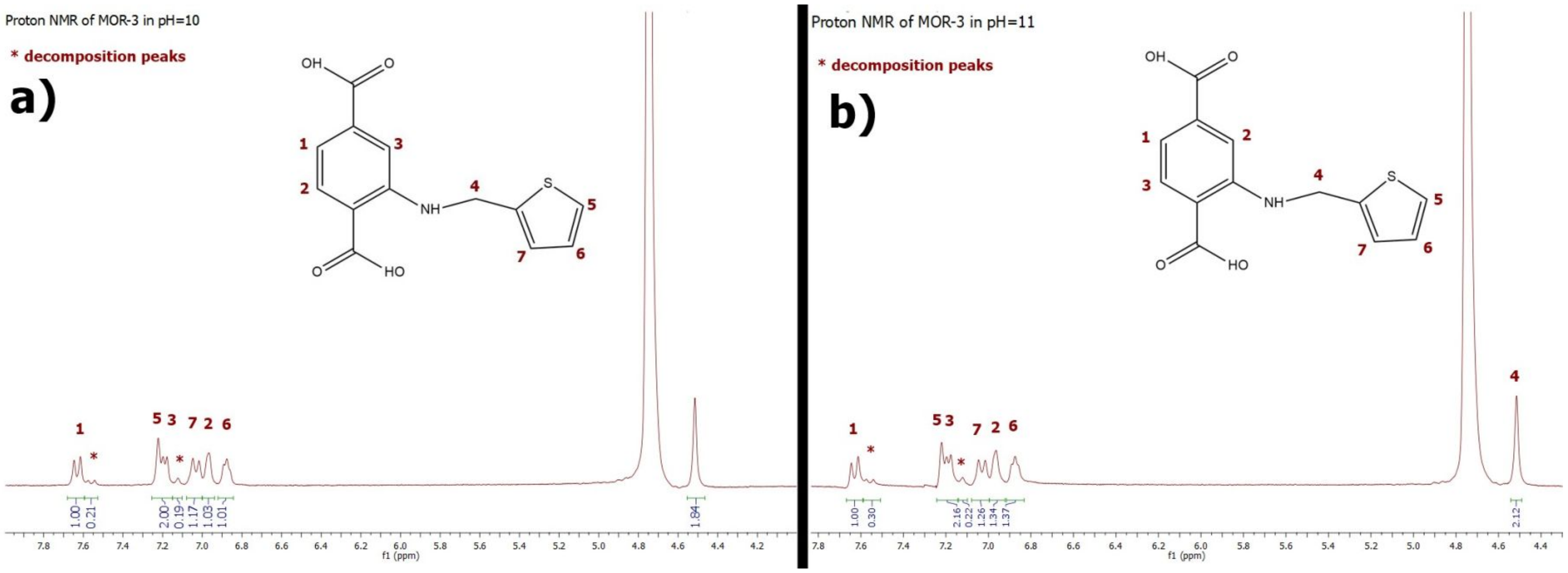

Figure S8. ^{1}H NMR spectrum of MOR-3 after its treatment with an aqueous solution at pH a) 10 and b)11 (the solid material was digested in 0.5mL D_2O/ 0.02g NaOH).

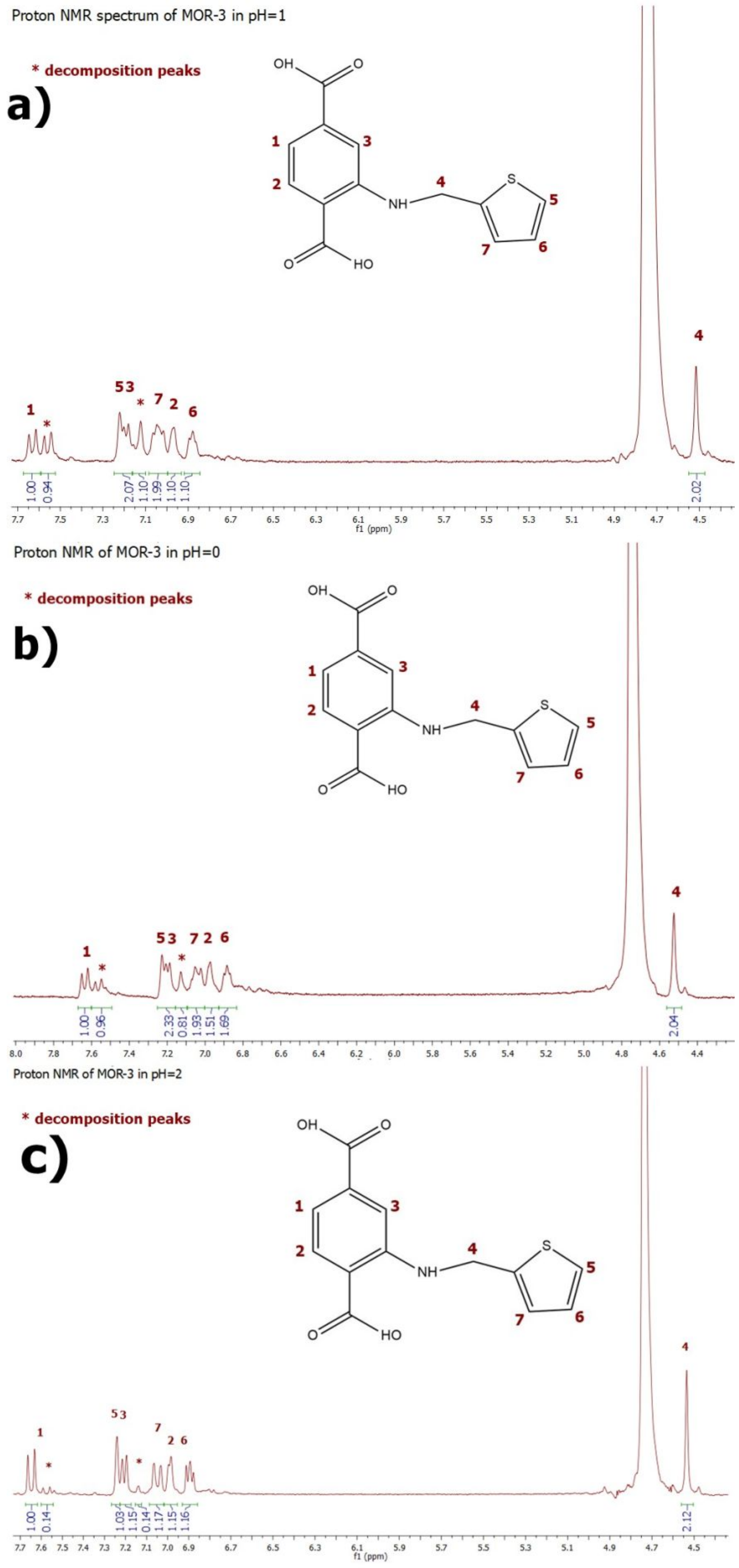

Figure S9. ^{1}H NMR spectrum of MOR-3after its treatment with an aqueous solution at pH a) 1, b) 0, and c) 2 (solid material was digested in 0.5mL D_2O/ 0.02g NaOH).

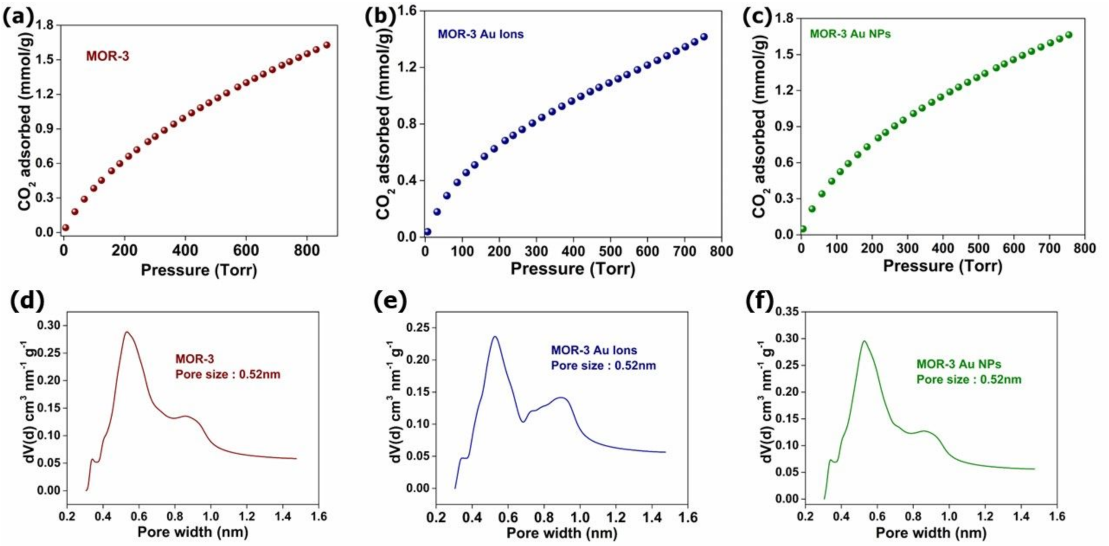

Figure S10. CO_2 adsorption isotherms at 273 K for (a) MOR-3, (b) MOR-3 Au Ions, and (c) MOR-3 Au NPs. Nonlocal density functional theory (NLDFT) micropore size distribution for (d) MOR-3, (e) MOR-3 Au Ions, and (f) MOR-3 AuNPs. The NLDFT analysis of the CO_2 adsorption data indicates pore sizes of 0.52 nm for MOR-3 before and after Au sorption.

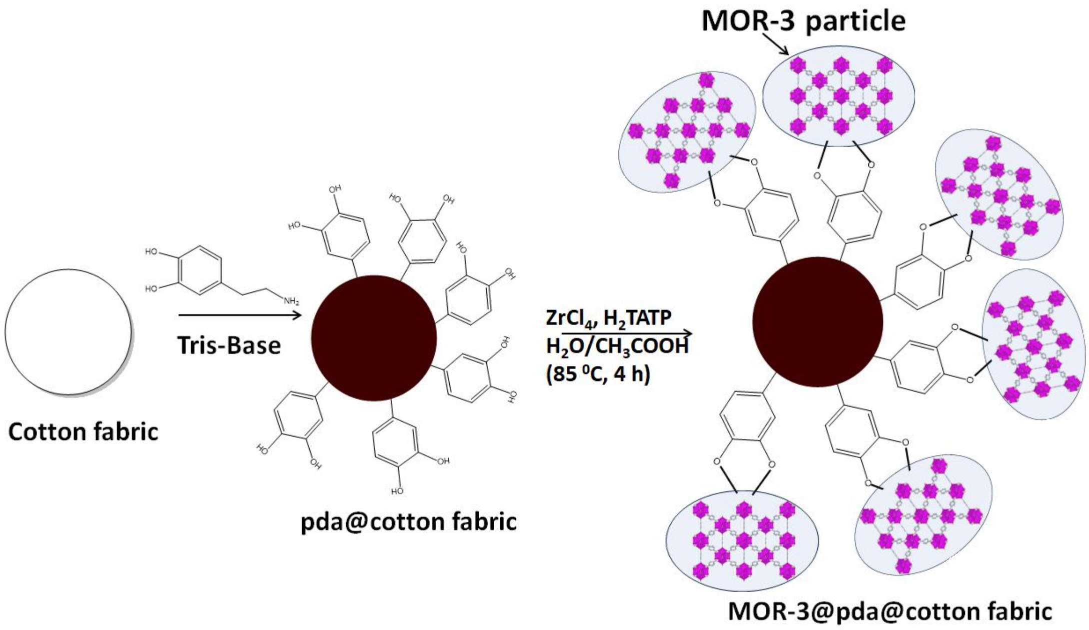

Scheme S2. Schematic representation of the cotton fabric pretreatment process and the subsequent immobilization of MOR-3 onto the matrix.

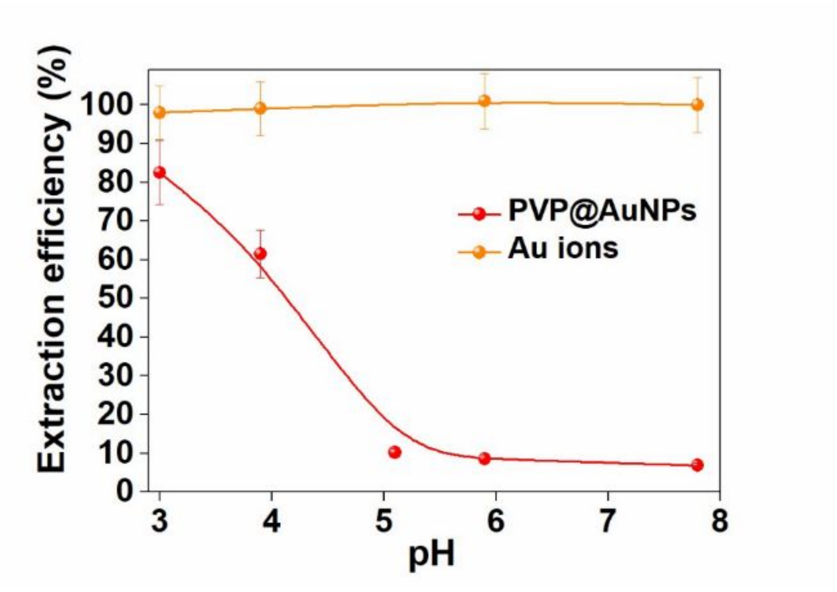

Figure S11. Extraction efficiency of Au Ions and PVP@AuNPs from MOR-3@pda@cotton fabric in aqueous solutions of various pH values.

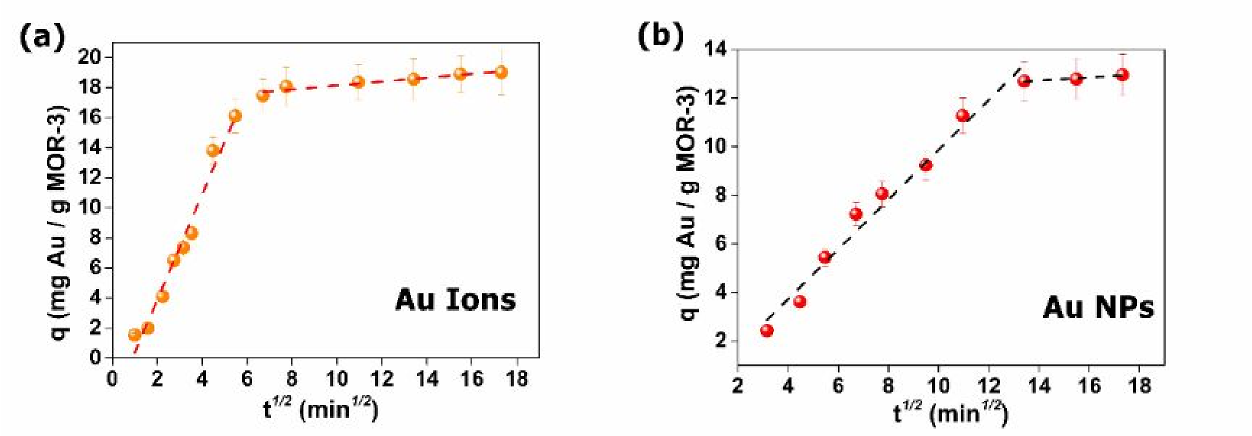

Figure S12. Webber-Morris plots for (a) Au Ions and (b) Au NPs sorption of MOR-3@pda@cotton fabric. Error bars represent the relative standard deviation.

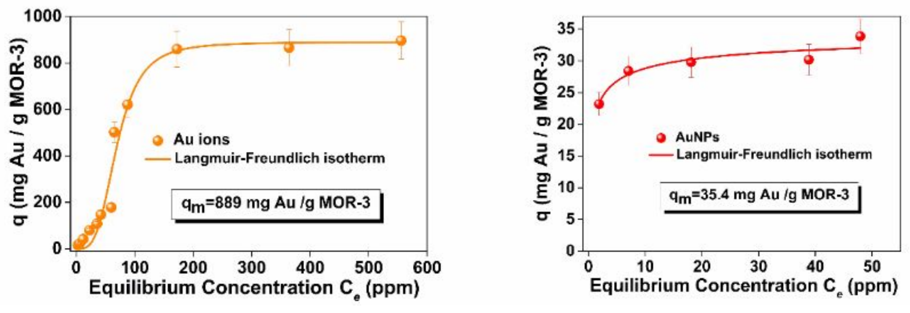

Figure S13. Fitting of the Langmuir-Freundlich isotherm model to the sorption of PVP@AuNPs and Au ions on MOR-3 powder.

The fitting of both Au ions and AuNPs isotherms was described by the Langmuir-Freundlich isotherm model, which is given as

$$q_e = q_m \frac{(bC_e)^{1/n}}{1+(bC_e)^{1/n}} \text{ ,}^{13}$$

where q_m indicates the maximum sorption capacity (in mg g^{-1}), b (L mg^{-1}) is the Langmuir constant related to the free energy of the sorption constant, n is the heterogeneous parameter, and C_e is the equilibrium concentration (mg L^{-1}). Fitting parameters are listed in detail in Table S1 below.

Table S1. Fitting parameters of Au isotherms of MOR-3@pda@cotton fabric

**Adsorbate**	**Langmuir-Freundlich**			
	**q_m (mg / g)**	**B (L / mg)**	**1/n**	**R^2**
Au NPs	43.392±1.113	7.096±0.142	0.104±0.021	0.982
Au Ions	883.48±63.24	0.046±0.012	0.723±0.073	0.990

Table S2. Fitting parameters of Au isotherms of MOR-3

**Adsorbate**	**Langmuir-Freundlich**			
	**q_m (mg / g)**	**B (L / mg)**	**1/n**	**R^2**
Au NPs	35.367±8.966	2.049±1.148	2.038±2.149	0.85
Au Ions	889.14±44.34	0.015±0.001	3.467±0.777	0.963

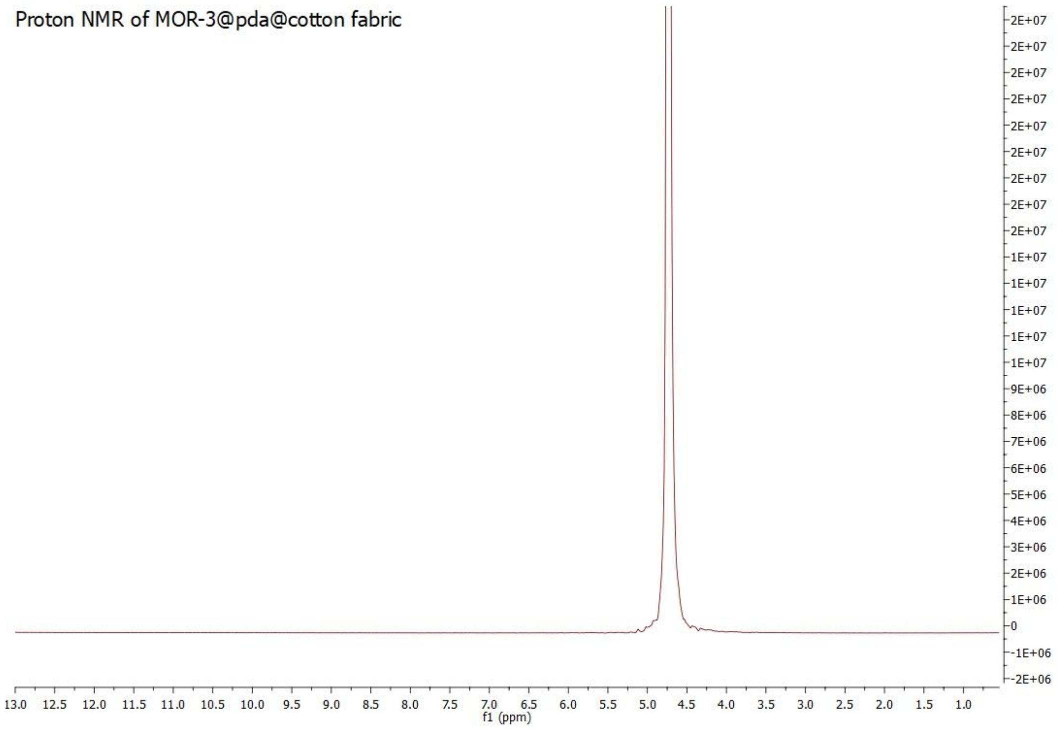

Figure S14. ^{1}H NMR of a water sample after MOR-3@pda@cotton fabric immersion. As can be seen, no solvent residues or organic ligands are detectable.

Table S3. Comparison of MOR-3@pda@cotton fabric with other sorbents for passive sampling applications.

Sampler	Inorganic species	Rs (mL/h)	Linear uptake phase time (days)	Reference
Chemcatcher	Cd	5.1	28	14
	Cu	4.9		
	Ni	5.7		
	Pb	0.7		
	Zn	5.3		
Chemcatcher	Cd	3.1	14	15
	Ni	2.7		
	Zn	2.9		

Chemcatcher	Rare earth elements	0.92-2.15	14	[16]
Chemcatcher	Hg	1.3-3.8	14	[17]
MOR-3@pda@cotton fabric	AuNPs	2.1	110	This work

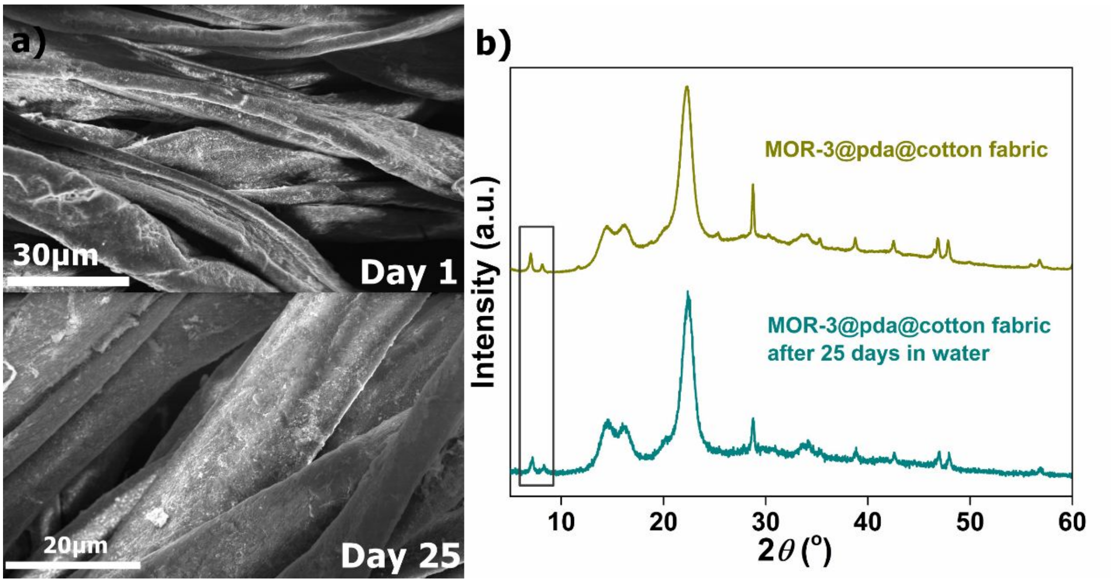

Figure S15. a) FE-SEM images and b) PXRD patterns of MOR-3@pda@cotton fabric before and after immersion of the fabric sorbent in water for 25 days.

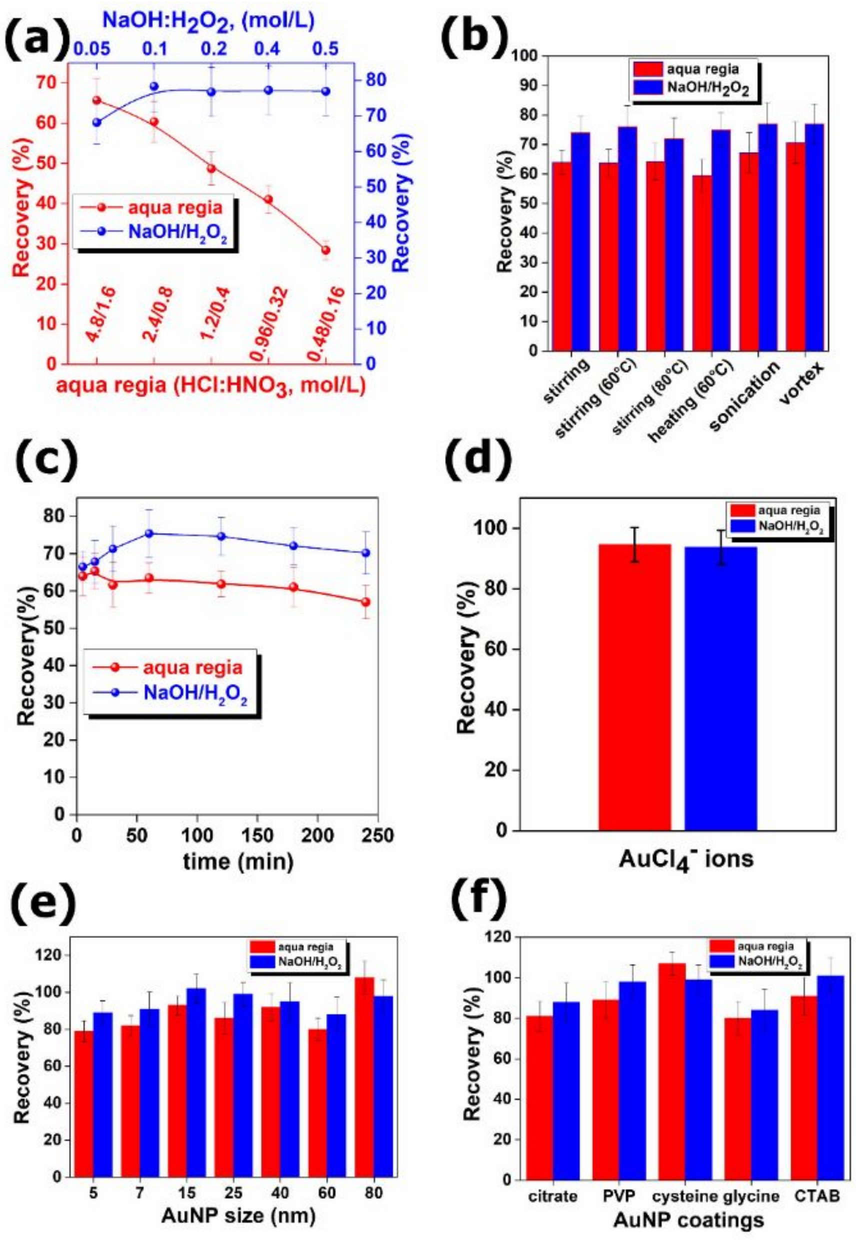

Figure S16. Optimization of experimental conditions for the elution of AuNPs from MOR-3@pda@cotton fabric. (a) Effect of elution solvent strength and composition (b) influence of elution time, (c) effect of elution method, (d) efficiency of eluting Au ions under the optimum conditions, and recovery of AuNPs of different (e) sizes and (f) surface coatings from MOR-3@pda@cotton fabric using (1:2) aqua regia. Recoveries were calculated compared to the determined sorption efficiency (Fig. 3).

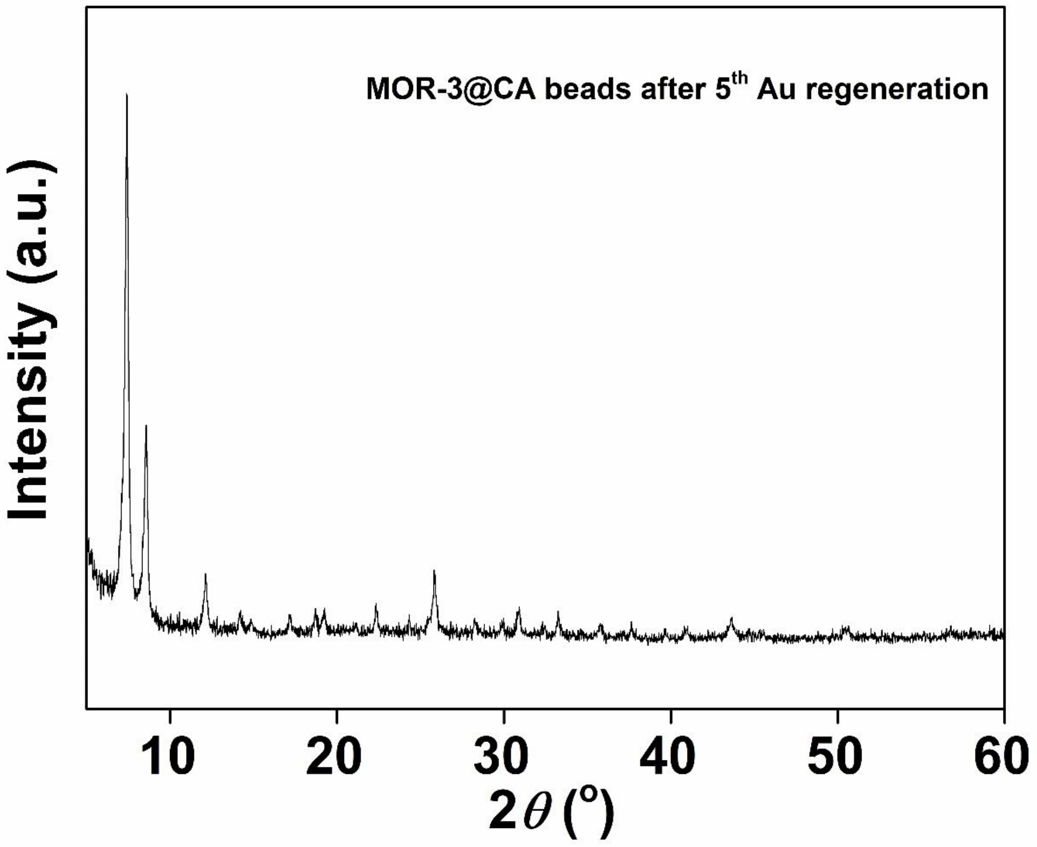

Figure S17. PXRD pattern of MOR-3@CA beads after 5th Au recovery with 1M acidic thiourea solution.

Table S4. Comparison of MOR-3@pda@cotton fabric with other state-of-the-art sorbents regarding AuNPs sorption properties.

Material	Physical Form	Au species	Maximum sorption capacity	Equilibrium time	Ref
Amine-rich cyclotriphosphazene (P3N3) nano-cages	Powder	Au(III)	1592 mg/g (pH=1.0, 60 °C)	≥10 h	18
Poly(acrylamide-co-acrylic acid) hydrogels	Gel	Au(III)	124 mg/g (pH=1)	72 h	19
Protein amyloid nanofibril aerogel	Gel	Au(III)	166.7 mg/g (pH<5)	30 min	20

Polymeric film	Film	Au(III)	0.4 µg/g (pH=7.4)	10 min	21
Guanidinium-based ionic covalent organic framework	Powder	Au(III)	1794 mg/g (pH~3)	100 min	22
Crystalline Hierarchical Porous MOP/COF aerogel	ionic gel	Au(III)	2349 mg/g (pH=3-5)	1 min	23
Polyethyleneimine (PEI)-functionalized paper	Paper	AuNPs	94 mg/g (pH=5)	24 h	24
Thiol and amine functionalized electrospun PVA membranes	membranes	AuNPs	84 mg/g (pH=6.5)	9 h	25
Co-polymer membrane-coated micrometer-sized carbon fiber aerogels	Gel	AuNPs	31.2 mg/g (pH= not reported)	>2.5 h	26
Mercaptosuccinate Zr(IV) MOF	Powder	Au(III), AuNPs	144 mg/g [Au(III)]<br>41.5 mg/g [AuNPs]<br>(pH = 6.5)	5 min (Au)<br>1 h (AuNPs)	27
Amino-Thiophene Zr(IV) MOF	cotton sheets	Au(III), AuNPs	883.5 mg/g [Au(III)] (pH=3-8)<br>43.4mg/ g [AuNPs] (pH =3)	1h (Au)<br>3 h (AuNPs)	This work
Amino-Thiophene Zr(IV) MOF	Powder	Au(III), AuNPs	889 mg/g [Au(III)] (pH=3-8)<br>35.4 mg/g [AuNPs] (pH =3)	10 min (Au)<br>3 h (AuNPs)	This work

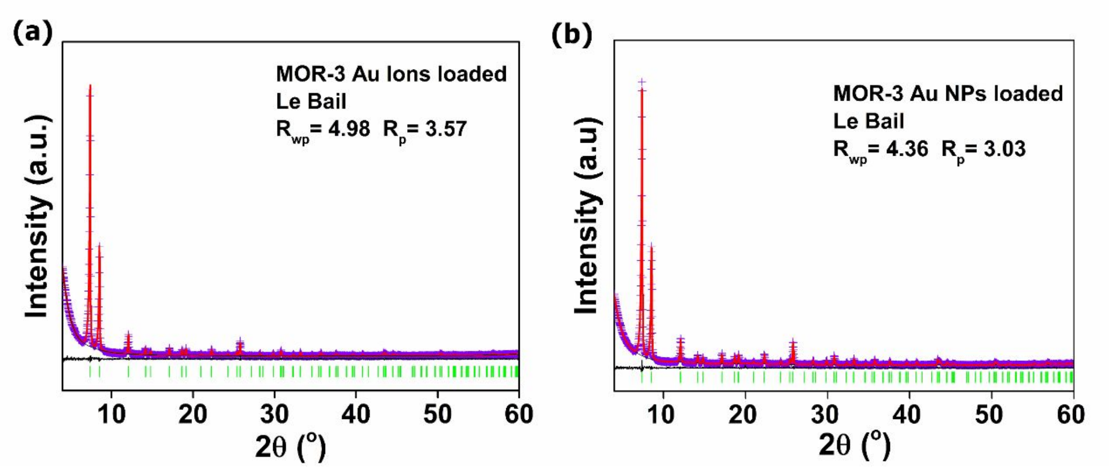

Figure S18. Le Bail plot of MOR-3 a) Au Ions and b) Au NPs loaded material. Violet crosses: experimental points; Red line: calculated pattern; Black line: difference pattern (exp. – calc.); Green bars: Bragg positions. Refined unit cell parameters for MOR-3 Au Ions: I4/m, a =14.675(1) Å, c= 20.720(3) Å, V= 4462(1) Å^3. Refined unit cell parameters for MOR-3 Au NPs: I4/m, a =14.669(2) Å, c= 20.715(3) Å, V= 4457(1) Å^3.

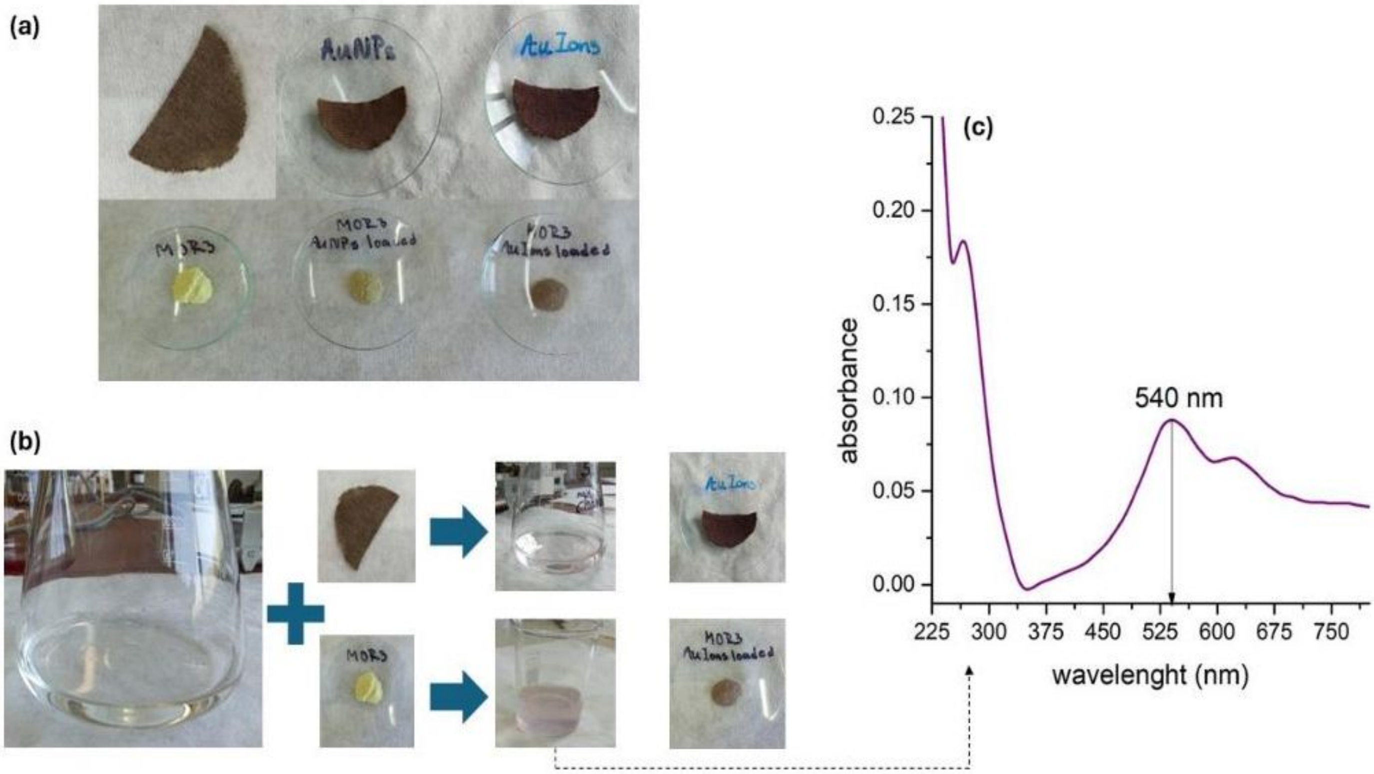

Figure S19. (a) MOR-3@pda@cotton fabric (upper image) and MOR-3 powder after sorption of PVP@AuNPs and $AuCl_4^-$ ions, (b) color of the solutions and the sorbents after sorption of $AuCl_4^-$ ions on MOR-3@pda@cotton fabric (upper image) and MOR-3 powder (lower image), (c) UV-Vis spectra of the aqueous solution after sorption of 50 mg/L of $AuCl_4^-$ ions on MOR-3 powder.

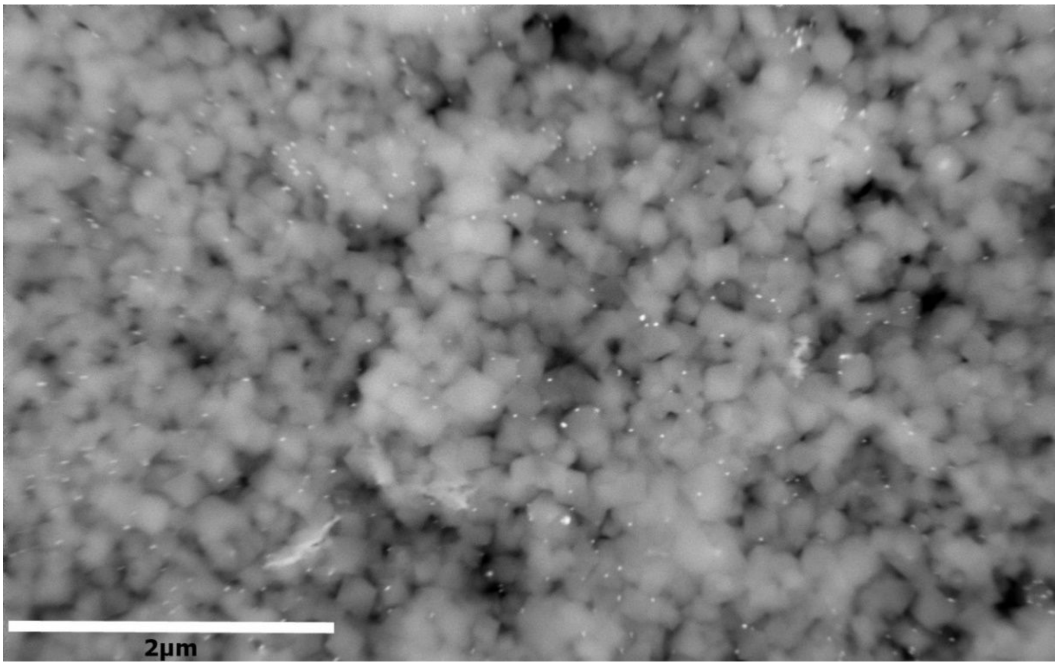

Figure S20. BSE-SEM image of Au NPs MOR-3 loaded material. The bright spots indicate Au NPs.

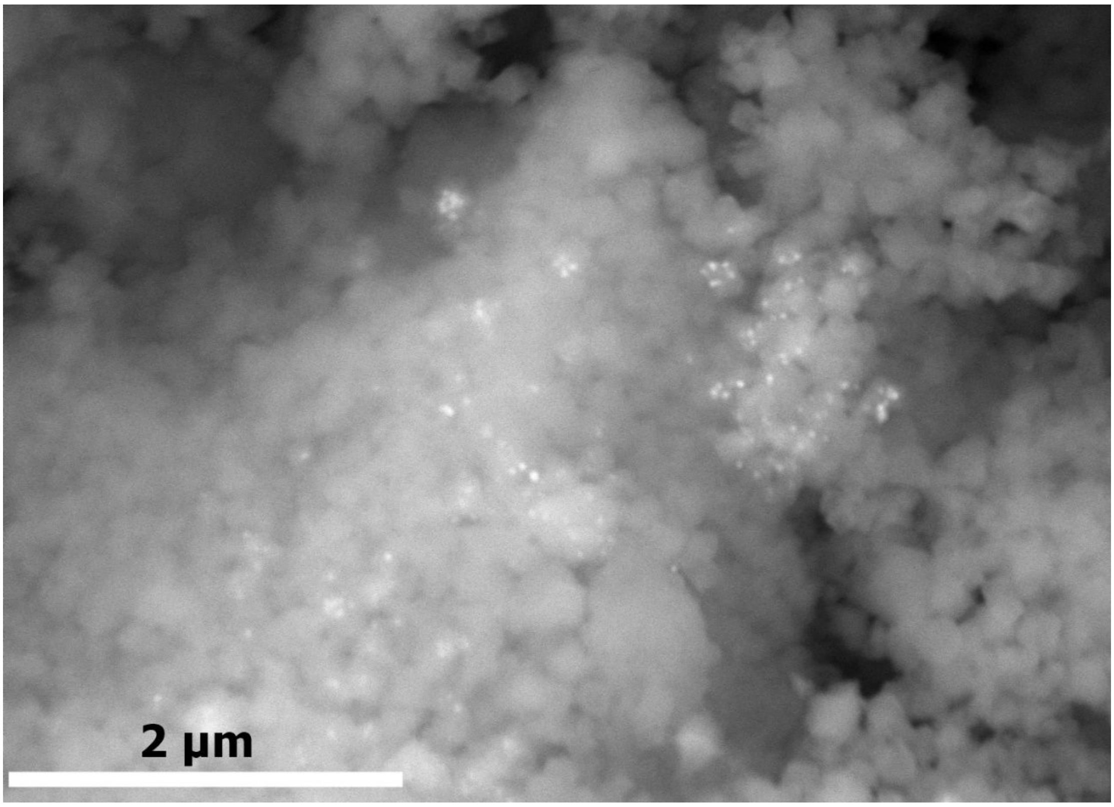

Figure S21. FE-SEM image of MOR-3@CA beads Au loaded material. The bright spots indicate Au NPs.

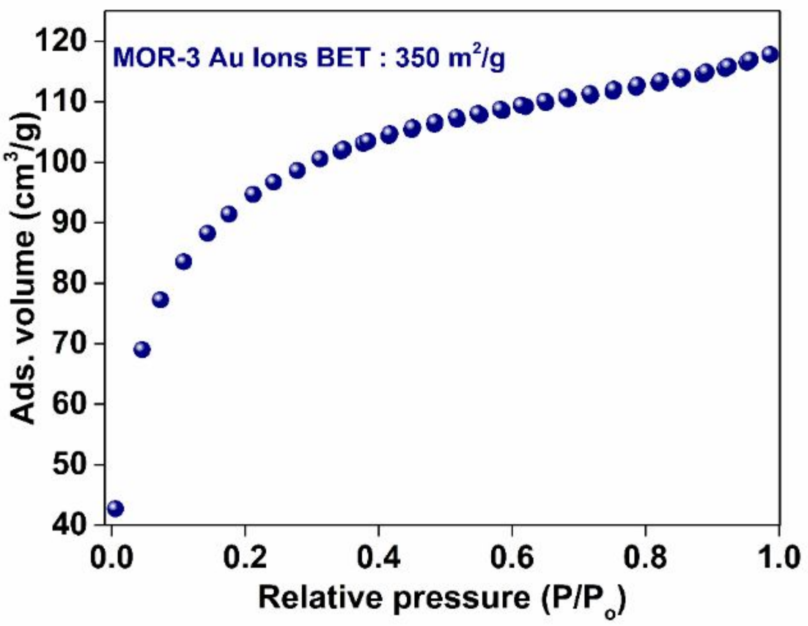

Figure S22. N_2 adsorption (77 K) for MOR-3 Au ions loaded material.

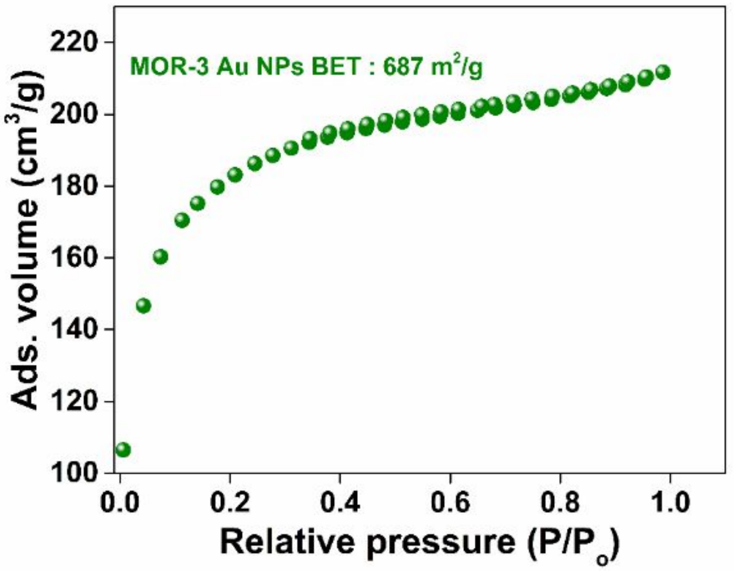

Figure S23. N_2 adsorption (77 K) for MOR-3 AuNPs loaded material.

Table S5. Binding energies of MOR-3 and Au loaded materials.

Material	Element	Orbital (core level)	Binding energy (eV)
**MOR-3**	N	1 s	398
	S	2 $p_{3/2}$	163.9
		2 $p_{1/2}$	162.6
	Zr	3 $d_{3/2}$	183.7
		3 $d_{5/2}$	181.3
**MOR-3 Au Ions**	N	1 s	398.1
	S	2 $p_{3/2}$	163.9
		2 $p_{1/2}$	162.6
	Zr	3 $d_{3/2}$	183.9
		3 $d_{5/2}$	181.5
	Au	4 $f_{5/2}$	86.7
		4 $f_{7/2}$	83
**MOR-3 Au NPs**	N	1 s	398.5
	S	2 $p_{3/2}$	163.9
		2 $p_{1/2}$	162.7
	Zr	3 $d_{3/2}$	184
		3 $d_{5/2}$	181.7
	Au	4 $f_{5/2}$	86.9
		4 $f_{7/2}$	83.1

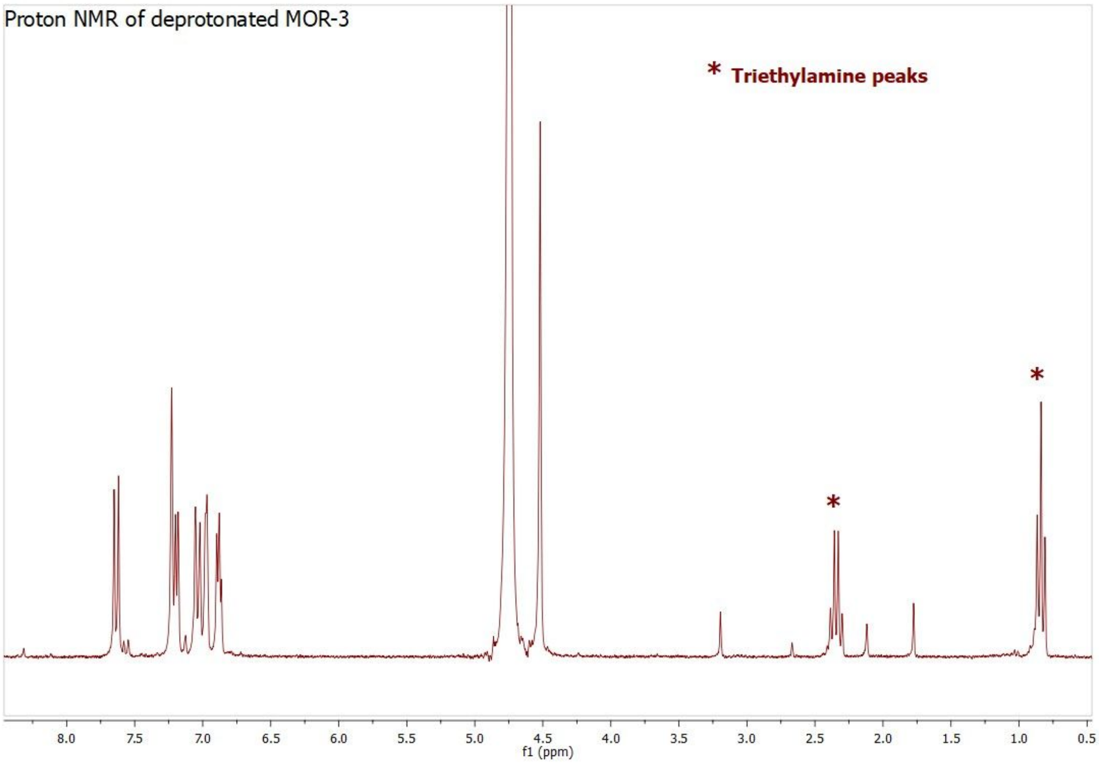

Figure S25. ^{1}H NMR of MOR-3 after treatment with a methanolic solution of triethylamine. Labeled triethylamine peaks are observed at 0.84 and 2.35 ppm.

References

(1) Ito, S.; White, F. J.; Okunishi, E.; Aoyama, Y.; Yamano, A.; Sato, H.; Ferrara, J. D.; Jasnowski, M.; Meyer, M. Structure Determination of Small Molecule Compounds by an Electron Diffractometer for 3D ED/ Micro ED. *Cryst. Eng. Comm.* **2021**, *23* (48), 8622–8630.

(2) Truong, K. N.; Ito, S.; Wojciechowski, J. M.; Göb, C. R.; Schürmann, C. J.; Yamano, A.; Del Campo, M.; Okunishi, E.; Aoyama, Y.; Mihira, T.; Hosogi, N.; Benet-Buchholz, J.; Escudero-Adán, E. C.; White, F. J.; Ferrara, J. D.; Bücker, R. Making the Most of 3D Electron Diffraction: Best Practices to Handle a New Tool. *Symmetry.* **2023**, *15* (8), 1555.

(3) Sheldrick, G. M. A Short History of SHELX. *Acta Crystallogr. A*. 2008, *64* (1), 112–122.

(4) Dolomanov, O. V.; Bourhis, L. J.; Gildea, R. J.; Howard, J. A. K.; Puschmann, H. OLEX2: A Complete Structure Solution, Refinement and Analysis Program. *J. Appl. Crystallogr.* **2009**, *42* (2), 339–341.

(5) Huang X. Gold Nanoparticles Used in Cancer Cell Diagnostics, Selective Photothermal Therapy and Catalysis of NADH Oxidation Reaction. Ph.D. Dissertation, Georgia Institute of Technology, **2006**.

(6) Vecchio, G.; Galeone, A.; Brunetti, V.; Maiorano, G.; Sabella, S.; Cingolani, R.; Pompa, P. P. Concentration-Dependent, Size-Independent Toxicity of Citrate Capped AuNPs in Drosophila Melanogaster. *PLOS One.* **2012**, *7* (1), e29980.

(7) Kapakoglou, N. I.; Giokas, D. L.; Tsogas, G. Z.; Ladavos, A. K.; Vlessidis, A. G. Development of a Chromium Speciation Probe Based on Morphology-Dependent Aggregation of Polymerized Vesicle-Functionalized Gold Nanoparticles. *Analyst.* **2009**, *134* (12), 2475–2483.

(8) Hartmann, G.; Baumgartner, T.; Schuster, M. Influence of Particle Coating and Matrix Constituents on the Cloud Point Extraction Efficiency of Silver Nanoparticles (Ag-NPs) and Application for Monitoring the Formation of Ag-NPs from Ag^+. *Anal. Chem.* **2014**, *86* (1), 790–796.

(9) Mandyla, S. P.; Tsogas, G. Z.; Vlessidis, A. G.; Giokas, D. L. Determination of Gold Nanoparticles in Environmental Water Samples by Second-Order Optical Scattering Using Dithiotreitol-Functionalized CdS Quantum Dots after Cloud Point Extraction. *J. Hazard. Mater.* **2017**, *323*, 67–74.

(10) Haiss, W.; Thanh, N. T. K.; Aveyard, J.; Fernig, D. G. Determination of Size and Concentration of Gold Nanoparticles from UV-Vis Spectra. *Anal. Chem.* **2007**, *79* (11), 4215–4221.

(11) Morin, N.; Miège, C.; Coquery, M.; Randon, J. Chemical Calibration, Performance, Validation and Applications of the Polar Organic Chemical Integrative Sampler (POCIS) in Aquatic Environments. *TrAC - Trends Anal. Chem*. **2012**, *36*, 144–175.

(12) Klet, R. C.; Liu, Y.; Wang, T. C.; Hupp, J. T.; Farha, O. K. Evaluation of Brønsted Acidity and Proton Topology in Zr- and Hf-Based Metal–Organic Frameworks Using Potentiometric Acid–Base Titration. *J. Mater. Chem. A.* **2016**, *4* (4), 1479–1485.

(13) Manos, M. J.; Kanatzidis, M. G. Sequestration of Heavy Metals from Water with Layered Metal Sulfides. *Chem. Eur. J.* **2009**, *15* (19), 4779–4784.

(14) Allan, I. J.; Knutsson, J.; Guigues, N.; Mills, G. A.; Fouillac, A.-M.; Greenwood, R. Chemcatcher® and DGT Passive Sampling Devices for Regulatory Monitoring of Trace Metals in Surface Water. *J. Environ. Monit.* **2008**, *10* (7), 821–829.

(15) Knutsson, J.; Knutsson, P.; Rauch, S.; Pettersson, T. J. R.; Morrison, G. M. Evaluation of a Passive Sampler for the Speciation of Metals in Urban Runoff Water. *Environ. Sci. Processes.* **2013**, *15* (12), 2233–2239.

(16) Petersen, J.; Pröfrock, D.; Paschke, A.; Broekaert, J. A. C.; Prange, A. Laboratory Calibration and Field Testing of the Chemcatcher-Metal for Trace Levels of Rare Earth Elements in Estuarine Waters. *Environ. Sci. Pollut. Res* **2015**, *22* (20), 16051–16059.

(17) Aguilar-Martínez, R.; Gómez-Gómez, M. M.; Palacios-Corvillo, M. A. Mercury and Organotin Compounds Monitoring in Fresh and Marine Waters across Europe by Chemcatcher Passive Sampler. *Int. J. Environ. Anal. Chem.* **2011**, *91* (11), 1100–1116.

(18) Abbas, Y.; Ali, S.; Ali, S.; Zuhra, Z.; Wang, H.; Bououdina, M.; Sun, Z. Amine-Rich Cyclotriphosphazene (P3N3) Nano-Cages for Enhanced and Selective Au(III) and Pd(II) Recovery and Hydrogen Generation: Waste-to-Resource Tactics. *Chem. Eng. J.* **2024**, *492*, 152127.

(19) Cinfrignini, P.; Boschetti, A.; Ghini, G.; Tenti, A.; Plazanet, M.; Martella, D.; Torre, R. A Gold Rush: Designing Hydrogels for Selective Recovery in Wastewater Containing Mixed Metal Ions. *ACS Appl. Mater. Interfaces.* **2024** *16* (49), 68368-68378.

(20) Peydayesh, M.; Boschi, E.; Donat, F.; Mezzenga, R. Gold Recovery from E-Waste by Food-Waste Amyloid Aerogels. *Adv. Mater.* **2024**, *36* (19), 2310642.

(21) Guo, W.; Liu, J.; Tao, H.; Meng, J.; Yang, J.; Shuai, Q.; Asakura, Y.; Huang, L.; Yamauchi, Y. Covalent Organic Framework Nanoarchitectonics: Recent Advances for Precious Metal Recovery. *Adv. Mater*. **2024**, *36*, 2405399.

(22) Xu, Q.; Du, X. H.; Luo, D.; Strømme, M.; Zhang, Q. F.; Xu, C. Gold Recovery from E-Waste Using Freestanding Nanopapers of Cellulose and Ionic Covalent Organic Frameworks. *Chem. Eng. J.* **2023**, *458*, 141498.

(23) Majumder, D.; Fajal, S.; Shirolkar, M. M.; Torris, A.; Banyla, Y.; Biswas, K.; Rasaily, S.; Ghosh, S. K. Nano-Springe Enriched Hierarchical Porous MOP/COF Hybrid Aerogel: Efficient Recovery of Gold from Electronic Waste. *Angew. Chem. Int. Ed.* **2025**,*64*, e202419830.

(24) Setyono, D.; Valiyaveettil, S. Functionalized Paper-A Readily Accessible Adsorbent for Removal of Dissolved Heavy Metal Salts and Nanoparticles from Water. *J. Hazard. Mater.* **2016**, *302*, 120–128.

(25) Mahanta, N.; Valiyaveettil, S. Surface Modified Electrospun Poly(Vinyl Alcohol) Membranes for Extracting Nanoparticles from Water. *Nanoscale.* **2011**, *3* (11), 4625–4631.

(26) Liu, R. L.; Mao, S.; Wang, Y.; Wang, L.; Ge, Y. H.; Xu, X. Y.; Fu, Q. A Mussel-Inspired Hybrid Copolymer Adhered to Chitosan-Coated Micro-Sized Carbon Fiber Aerogels for Highly Efficient Nanoparticle Scavenging. *Environ. Sci. Nano.* **2017**, *4* (11), 2164–2174.

(27) Tziasiou, C.; Andreou, E. K.; Armatas, G. S.; Manos, M. J.; Pournara, A. D.; Giokas, D. L. Zr^{4+}-Mercaptosuccinate MOF for the Uptake and Recovery of Gold Nanoparticles and Gold Ions under Batch and Continuous Flow Conditions. *Chem. Eng. J.***2024**, *489*, 151107.
